# Rising tones and rustling noises: Metaphors in gestural depictions of sounds

**DOI:** 10.1371/journal.pone.0181786

**Published:** 2017-07-27

**Authors:** Guillaume Lemaitre, Hugo Scurto, Jules Françoise, Frédéric Bevilacqua, Olivier Houix, Patrick Susini

**Affiliations:** 1 Equipe Perception et Design Sonores, STMS-IRCAM-CNRS-UPMC, Institut de Recherche et de Coordination Acoustique Musique, Paris, France; 2 Equipe Interaction Sons Musique Mouvement, STMS-IRCAM-CNRS-UPMC, Institut de Recherche et de Coordination Acoustique Musique, Paris, France; Tokyo Daigaku, JAPAN

## Abstract

Communicating an auditory experience with words is a difficult task and, in consequence, people often rely on imitative non-verbal vocalizations and gestures. This work explored the combination of such vocalizations and gestures to communicate auditory sensations and representations elicited by non-vocal everyday sounds. Whereas our previous studies have analyzed vocal imitations, the present research focused on gestural depictions of sounds. To this end, two studies investigated the combination of gestures and non-verbal vocalizations. A first, observational study examined a set of vocal and gestural imitations of recordings of sounds representative of a typical everyday environment (ecological sounds) with manual annotations. A second, experimental study used non-ecological sounds whose parameters had been specifically designed to elicit the behaviors highlighted in the observational study, and used quantitative measures and inferential statistics. The results showed that these depicting gestures are based on systematic analogies between a referent sound, as interpreted by a receiver, and the visual aspects of the gestures: auditory-visual metaphors. The results also suggested a different role for vocalizations and gestures. Whereas the vocalizations reproduce all features of the referent sounds as faithfully as vocally possible, the gestures focus on one salient feature with metaphors based on auditory-visual correspondences. Both studies highlighted two metaphors consistently shared across participants: the spatial metaphor of pitch (mapping different pitches to different positions on the vertical dimension), and the rustling metaphor of random fluctuations (rapidly shaking of hands and fingers). We interpret these metaphors as the result of two kinds of representations elicited by sounds: auditory sensations (pitch and loudness) mapped to spatial position, and causal representations of the sound sources (e.g. rain drops, rustling leaves) pantomimed and embodied by the participants’ gestures.

## 1 Introduction

Consider this excerpt of a conversation between two persons describing a sound, (gestures are indicated between brackets) [1]:

- “It sounds like as if you would take a piece of corrugated cardboard *[grabbing an imaginary piece of cardboard]*. First you scrape it *[rubbing both hands]*, then you tear it off *[moving apart clenched fists, as tearing off an imaginary piece of cardboard in two pieces]* and it sounds like Rrrrrrrr off the cardboard *[right fist bouncing off his chest, as moved by an imaginary spring]*. You see? First, Ffffff *[palms extended, both arms moving inward, as if rubbing a flat surface]*, and then Rrrrrrrr *[clenching fists, right fist bouncing off his chest to an extended arm position]*.

- Oh I see: Ffffff then Rrrrrrr.”

As illustrated by this example, speakers often use non-verbal imitative vocalizations and gestures to describe sounds when they run out of words, as sounds are notably difficult to describe for lay persons [2–4]. In fact, imitative behaviors are widespread in humans [5–8], and we have recently shown that imitative vocalizations communicate sounds very effectively [9, 10].

Whereas our previous work focused on vocalizations, the current study investigates the gestures that accompany them. Potentially, such gestures could serve several functions (see below). For example, they could punctuate verbal communication (“beats”), help the physical production of vocalizations, or carry semantic information.

The importance of gestures during verbal communication has been acknowledged for a long time. Early on, Ekman and Friesen categorized gestures accompanying verbal communication as emblems (carrying meaning through shared symbols), illustrators (illustrating the meaning conveyed by speech), affect displays, regulators of the conversation, and adaptors [11]. Kendon also defined a continuum encompassing gesticulation, emblems, pantomime (carried out without speech), and sign language [12]. McNeill further defined four dimensions of gestures: iconic (e.g. a knocking gesture to signify knocking on a door), metaphoric (similar to iconic but for abstract ideas), deictic (pointing elements in space), and beats (taking the form of hands beating time along speech) [13].

For these authors, gestures and speech are co-expressive and intertwined, and gestures have a clear communicative role in conversations. In particular, gestures can provide effective demonstrations and indications because they illustrate more directly what they refer to [14–16], whereas speech is particularly well suited for concrete and abstract descriptions. For example, seeing teachers’ gestures improves the learning of mathematical concepts [17]. The communicative role of gestures may also be more subtle. For example, Krauss has argued that a fundamental role of gestures may be to aid lexical retrieval in speakers by activating the spatio-dynamic features of concepts [18, 19]. Goldin-Meadow and colleagues have also shown that teachers can interpret mismatches between speech and gestures when children explain how they solve a problem, and adjust accordingly their instructions to these children [14, 20].

The current study focuses on how people consciously *depict* a sound they have just heard (without seeing the physical production of the sound) with their arms, hands, etc. together with non-verbal vocalizations. The study excluded symbolic gestures and onomatopoeias to limit the influence of conventionalized signs and words. According to Clark [21], such depictions are based on physical analogs and systems of mappings between a proximal scene (the gestures and vocalizations performed by a person) and a distal scene (what the person is referring to). For the sake of consistency with our previous work, we simply call here the distal scene the “referent sound” (this might refer to more than the sound signal, even when imitators can only hear the sounds, see below), the person who is trying to communicate the referent sound the “imitator” (even though gestures cannot be considered as imitations, see below), and the person perceiving and decoding the gestural and vocal depictions the “receiver”.

According to Clark, depictions are selective (they cannot represent all parts of the referent sounds) and perceptually bound: there has to be some kind of correspondence between the gesture and the referent sound [21]. From the point of view of the receiver, gestures depicting sounds are visual signals. When listeners can only hear the sounds (and not see the scene), the task of gestures is to communicate *auditory* sensations and representations with *visual* signals. In comparison, vocal imitations of sounds translate acoustic features into some other acoustic (vocal) features [22, 23]. As such, gestures are probably better considered as illustrative ideographs [11], rather than imitations.

This idea of correspondence between the auditory and visual domains is puzzling. Let us first consider what listeners perceive. In fact, listeners can engage in two main “modes” of listening [24, 25]: musical (or reduced) and everyday (causal) listening. In the former case (musical listening), listeners concentrate on their auditory sensations, irrespectively of their source. But in most cases, listeners found it extremely difficult to disregard the physical source of the sounds [26]. Instead, they interpret the sources of the sounds, the context in which they are produced (“causal listening”), and make semantic associations (thus defining an additional “semantic” mode of listening [27]). More specifically, listeners can recover many properties of the sound sources: the size, shape, material of the vibrating objects [28–31], and the actions and the gestures that create the sounds [32–35]. Auditory representations thus include auditory sensations and representations cause the sounds.

To communicate a sound, imitators have therefore to establish some correspondence between the visual aspects of their gestures and the different possible representations evoked by sounds (auditory sensations and causal representations). As such gestures depicting sounds can be considered as metaphors, as defined by Lakoff and Johnson: “The essence of metaphor is understanding and experiencing one kind of thing in terms of another” ([36], [p. 5]). Such metaphors could first establish a correspondence between auditory sensations (pitch, loudness, timbre) and visual features. For example, Spence has reported a number of correspondences systematically found across a variety of tasks and paradigms: pitch is associated with visual elevation, brightness, lightness, shape, size, and spatial frequency; loudness is associated with brightness [37] (though such associations may interact a lot and be asymmetrical [38]; loudness is also associated with horizontal position [39]). In fact, the “spatial metaphor of pitch” is ubiquitous in the literature: listeners usually associate pitch with some spatial dimension. Most authors have considered the vertical dimension (sometimes confounded with depth), yet associations with horizontal position or thickness have also been reported [40–45]. Other authors have been able to replicate the effect of the vertical association only when pitch covaries congruently with brightness [46]. Parise et al. (2014) have related this association to the statistical properties of natural auditory scenes and characteristics of the human auditory system [47]. The spatial metaphor of pitch also shows up in many languages (for example“high” and “low” pitches in English) but is not the result of a language convention: the linguistic metaphor seems rather to be the result of a deeper cognitive association [48]. The association of pitch and vertical position has been shown to occur in infants as young as 3-4 months old [49], yet some other results suggest that such associations are not mature before an older age [50, 51].

Second, metaphors can also establish a correspondance between the depicting gestures and the causes of the sounds by pantomiming the causal situation that creates the sounds, and in particular the gestures that create the sounds. For example, Caramiaux and colleagues have shown that many people describing a sound with their hands (“sound tracing”, see below) actually pantomime the source of the sounds when they identify it (e.g. beating eggs) [52] (“motormimetic sketching” [53]). More generally, the idea that listeners perceive (and embody) the actions and gestures that create the sounds (i.e. embodied cognition) is at the core of the work of musicologists such as Marc Leman, who consider that music expresses corporeal intentions of the musicians or composers, which are perceived as actions by listeners [54–56]. Depicting sounds with gestures could thus consist in *embodying* the sounds: re-enacting the gestural representations elicited in listeners.

The idea of music embodiment has been empirically explored in “sound tracing” studies (usually motivated by the design of new digital musical instruments). In those studies, participants perform free movements that “correspond well” to the sounds they hear [57]. Apart from the already mentioned pantomiming, they have repeatedly highlighted the spatial metaphor of pitch [58–60], as well as correspondences between gesture velocity and loudness [61].

The goal of the present study was to explore the metaphorical gestures used in combination with non-verbal vocalizations to communicate the auditory sensations elicited by non-vocal everyday sounds. We conducted two complementary studies. In the first, observational study, we manually examined a set of vocal and gestural imitations (created with a communicative goal) of a large variety of everyday sound recordings. The set of sounds was ecologically valid (representative of sounds that occur in a typical everyday environment), with complex acoustic characteristics. These imitations were manually annotated. The results highlighted a few potential metaphors that a second, experimental study investigated in more depth. In this second experiment, we used non-ecological stimuli, with only a few acoustic parameters varying and specifically designed to elicit the behaviors highlighted during the observational study. We used quantitative measures of vocal and gestural features specifically designed to represent the potential metaphors used to convey different aspects of the gestures. Whereas sound tracing studies have been mostly motivated by the comparison of the temporal evolution of different acoustic and gestural features [52, 58, 62], the goal of these measures was to highlight metaphors not necessarily time-locked with the sound evolution [27]. Even though the main focus of this study is on gestures, we also analyzed the vocal productions of the imitators to get a full picture of what they intended to communicate. Data from these studies are available at https://doi.org/10.5281/zenodo.569486 and https://doi.org/10.5281/zenodo.804038.

## 2 Observational study

The first, observational study analyzed the manual annotations of a set of vocal and gestural imitations of a selection of everyday sounds. This set was selected from a larger database (50 imitators imitating 52 sounds under different instructions), which was part of a larger project [63]. For the current study, we randomly selected a subset of ten imitators imitating eight referent sounds. This selection limited the size of the data (80 audio and video recordings) to make the annotation tractable by human annotators. The coauthors annotated the database to highlight the characteristics of the vocalizations and gestures used to imitate the sounds. The annotation consisted in selecting descriptions from a list of possible voice and gestures phenomena, elaborated during preliminary observations of the database. The goal of this study was to qualitatively describe the features of the gestures and vocalizations used by the imitators.

### 2.1 Material: The referent sounds

The referent sounds used in the observational study were produced by events and products commonly occurring in an everyday environement. They were selected from our previous studies [26, 33, 64, 65], commercial or freely available databases (Hollywood Edge, Blue Box, Sound Ideas, Freesound, etc.), as well as recorded from human computer interfaces (mobile phones, video games, computer operating systems) or synthesized.

Our previous studies and pilot observations revealed that two aspects of the referent sounds have an important influence on the imitations: *tonalness* and *morphological profile* [22]. Tonalness refers to the magnitude of the sensation of pitch in a sound (from a weak sensation of pitch to a strong sensation of pitch; tonalness is similar to “pitch strength” [66], see [67] for a discussion). Pure tones and harmonic complexes are very tonal whereas random noises are non-tonal (i.e. noisy). Sounds that combine at the same time tonal and noisy components, inharmonic complexes, and narrow-band noises elicit tonalness sensations between pure tones and broadband noises. Here, we used sounds that elicited a strong sensation of pitch (tonal), a very weak sensation of pitch (noisy), or combined tonal and noisy elements in time. Morphological profiles describe the temporal evolution of the acoustic properties of sound. Typical examples of morphological profiles are ascending or descending pitches (i.e. upward and downward sweeps) [68]. The selection balanced discrete (impulsive, repeated) and continuous profiles (continuous sequences, etc.).

**Fig 1:**
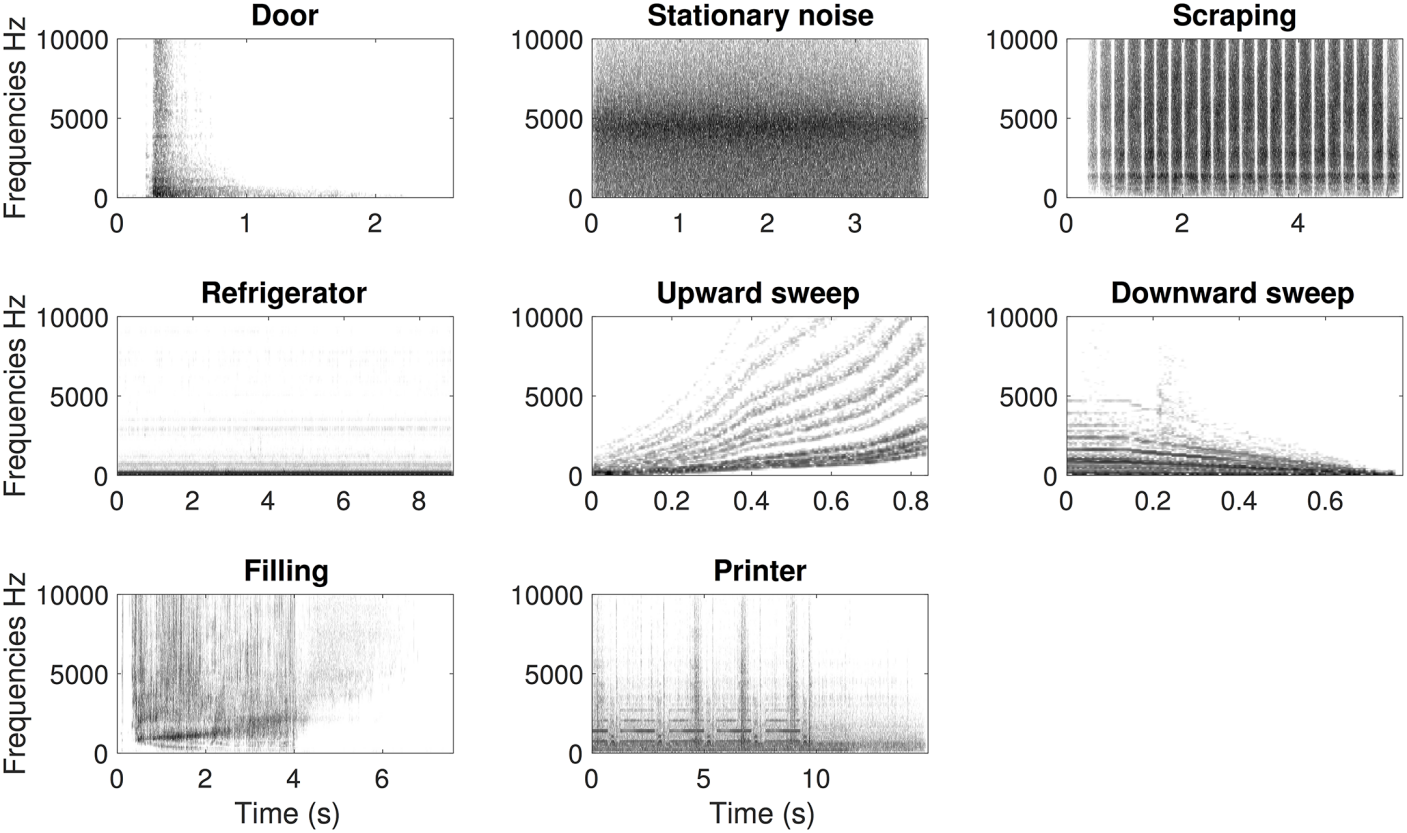
Spectrograms of the eight referent sounds used in the observational studies. The frequency range has been limited to 10kHz for the sake of legibility.

The combination of tonalness and morphological profiles created eight referent sounds, summarized in Table 1 and represented in Fig 1. These eight sounds can be described as simple noisy sounds, simple tonal sounds, and complex combinations of noisy and tonal elements.

**Table 1:** The selection of referent sounds used in the observational study.

Referent sound	Tonalness	Morphology	Description
		Simple noisy sounds	
Door	Noisy	Impulsive	Slamming a door
Stationary noise	Noisy	Stationary	A stationary noisy sequence
Scraping	Noisy	Repeated	Smoothing a piece of wood with sandpaper
		Simple tonal sounds	
Refrigerator	Tonal	Stationary	A low hum with some quiet vibrations
Upward sweep	Tonal	Upward	A harmonic sound with an upward pitch
Downward sweep	Tonal	Downward	A harmonic sound with a downward pitch
		Combined sounds	
Filling	Combined	Complex	Filling a receptacle with sparkling water
Printer	Combined	Complex	A printer printing out pages

### 2.2 Methods: Recording and annotating imitations

We recorded and selected *vocal and gestural imitations* of referent sounds performed by human imitators. The imitations were analyzed by four of the coauthors, who watched each video and annotated them to describe the main characteristics of the vocalizations and gestures.

#### 2.2.1 Recordings

Overall, the procedure consisted in listening to the referent sounds and recording an imitation. The database of vocal and gestural imitations has been registered with the French National Commission on Informatics and Liberty (CNIL 1890949).

**Setup**

The imitators were seated in a double-walled IAC sound isolated booth. The setup consisted of a microphone (DPA d:fine omni), an audio interface (RME Fireface 800), a pair of studio monitors (Yamaha MSP5), and an Apple Mac Pro with Intel Dualcore 2.6 GHz, running MacOS 10.6.8 to record the vocal imitations. It also included a high-definition video camera (GoPro Hero4), and LED located in the cameras’ field of view (to be used as a synchronization signal), and a micro-controller (Arduino) controlling the LED. The recording of the video and the triggering of the LED signal was managed by an Apple MacMini desktop computer (Intel Quadcore 3.2 GHz) running MacOS 10.9. The setup also included two inertial motion units (IMU, Ircam’s “Musical Objects”: MO) fixed on the participants’ wrists. Each IMU contains 3D accelerometers and 3-axis gyroscopes and transmits the data wirelessly with a latency of about 5-10 ms [69]. The recording of the IMU data was managed by an Asus PC with Intel Quadcore 3.2 GHz, running Microsoft Windows 8.

The user interface and data stream management were fully developed in Max/MSP v.6.1 (Ircam/Cycling74), using Ircam’s Mubu Max/MSP externals for the IMUs (http://forumnet.ircam.fr/fr/product/mubu/, last retrieved on August 17, 2015). We used Harald Meyer’s GoPro Camera Control 2 for the wifi control of the GoPro camera (http://www.tequnique.com/gopro, last retrieved on August 18, 2015).

The audio was recorded at a sampling rate of 64 kHz, in 16 bits PCM WAV files, the video at 120 frames per second (1920 x 1080 pixels). Data from the IMUs were collected at 100 Hz.

To allow a precise synchronization of the different files during post-production, a multimedia synchronization signal was generated at the same time, 1500 ms after the participant initiated the recording, with the LED flashing a red signal, a sampled “clap” sound fed to the audio tracks, and a vector of arbitrary numbers for the motion and gesture data.

**2.2.1.1 Imitators.** Ten imitators (five male, five female) aged from 20 to 40 years old (median age 26 years old) were randomly selected for this study from an original pool of 50 persons. All reported normal hearing and were native speakers of French. None of them had received formal training in music, audio, dance, theater, or sign language. None of the imitators had any practice of vocal imitation or Foley artistry.

**2.2.1.2 Procedure.** Whereas the goal of this study was to explore how listeners *communicate* sounds, our previous work with face-to-face conversations highlighted that such conversations involve a lot of negotiation between the speakers, and that these negotiations also rely on gestures [1]. To prevent such social gestural interactions to interfere with the communicative gestures we were interested in, the procedure consisted in having imitators record vocal and gestural imitations to a computer interface, with the instruction to record such productions for a future experiment with another person. The instructions specified that someone would listen or watch their imitations and would have to identify the sounds and retrieve them from a list of distractors. The user interface allowed the imitators to listen to the referent sounds, record and play back an imitation. Imitators could record an imitation only if they had listened to the referent sound at least once. The imitators were autonomous during the experiment to enable maximum creativity without being intimidated by the presence of the experimenter. They were instructed not to use any conventional onomatopoeia. Because we were interested in how people describe the sounds themselves (and not the source of the sounds or the situation in which the sounds occur), all imitators were also instructed not to pantomime the situation, to encourage musical listening. The order of the sounds on the interface was randomized for each imitator. The experimental interface presented the referent sounds and the imitations on the same interface, so that the imitators could compare the different sounds. The imitators were encouraged to compare and evaluate the quality of their imitations, and to compare their imitations with the referent sounds. Imitators were instructed to record their imitations until they were satisfied (within the limit of five trials). Accordingly, we considered only the last imitation. After the recording session, the experimenter reviewed all the recordings with the imitators and the imitators were invited to comment their imitations and explain their strategy (“self-confrontration interview” [52, 70]). The self-confrontation interview was recorded for later analysis.

#### 2.2.2 Annotations

**2.2.2.1 Annotators.** Four of the coauthors annotated the selection of imitations independently.

**2.2.2.2 Procedure.** The annotators annotated each video file using three annotation fields. For each of these fields, the annotator selected one response among a list of possible responses (see Table A in S1 Appendix). The annotators also had the possibility to create an ad-hoc response when none of the proposed responses fitted their impression. The annotation fields and the different possible annotations had been created based on pilot observations of the whole database and previous work [22]. The annotators were aware of which referent sound each imitation referred to.

The annotation fields were:

- Main vocal feature(s): the annotators described what they perceived as being the most important features of the vocalizations that were characteristic of the referent sounds. They could indicate that the vocalization was tonal or noisy, that the pitch or the spectral centroid of the voice was rising up or down, that it contained some spectral modulation, roughness, or some modulation created by breathing in and out.
- Main gestural feature(s): the annotators described what they perceived as being the most important features of the gestures that were characteristic of the referent sounds. For example, they could indicate that the imitators were raising or lowering their hands, opening or closing their fingers, shaking their hands or fingers, getting their hands closer or farther away, etc.
- Main direction(s) of the gesture: the annotators indicated the main direction(s) of the gestures: upward or downward, inward or outward, leftward or rightward.

The annotators made these annotations globally for each imitation, and without segmenting the imitations in different phases or annotating the timing of the different elements. In general, imitations were short (about a few seconds long).

Eventually, the annotators compared their annotations. Whenever they disagreed, the imitation was carefully reexamined and a decision was made to keep both annotations or remove one. This produced a reconciled annotation that was used for the next analyses. The agreement between annotators was assessed by computing Fleiss’ *κ* for each response field [71]. Because annotators could provide several responses for each sound, the procedure was adapted: each possible response was coded as a dummy binary variable; *κ* values were computed for each possible response, then averaged across all possible responses. Agreement was *κ* = 0.56 for the main vocal features (moderate agreement), *κ* = 0.41 for the main gestural features (moderate agreement), and *κ* = 0.65 for the main direction of the gestures (substantial agreement).

### 2.3 Results and interpretation

The annotations were first analyzed by counting the occurrences of each annotated feature for the different referent sounds. Then the analyses focused on co-occurrences of vocal and gestural features. These analyses were finally compared to what the imitators described during the self-confrontation.

#### 2.3.1 Occurrences

The following paragraphs describe the vocal and gestural strategies used by the imitators for each referent sound. For the sake of clarity, we only report the features that occurred in more than 60% of the imitations. The full data are reported in Tables B to D in S1 Appendix.

**2.3.1.1 Simple tonal sounds.** As expected, most imitators produced tonal vocalizations to imitate the tonal referent sounds (90% for the upward sweep, 100% for the downward sweep, 100% for the refrigerator, sometimes combined with a noisy component). All of them produced a vocalization with a pitch motion consistent with the referent sounds.

**Fig 2:**
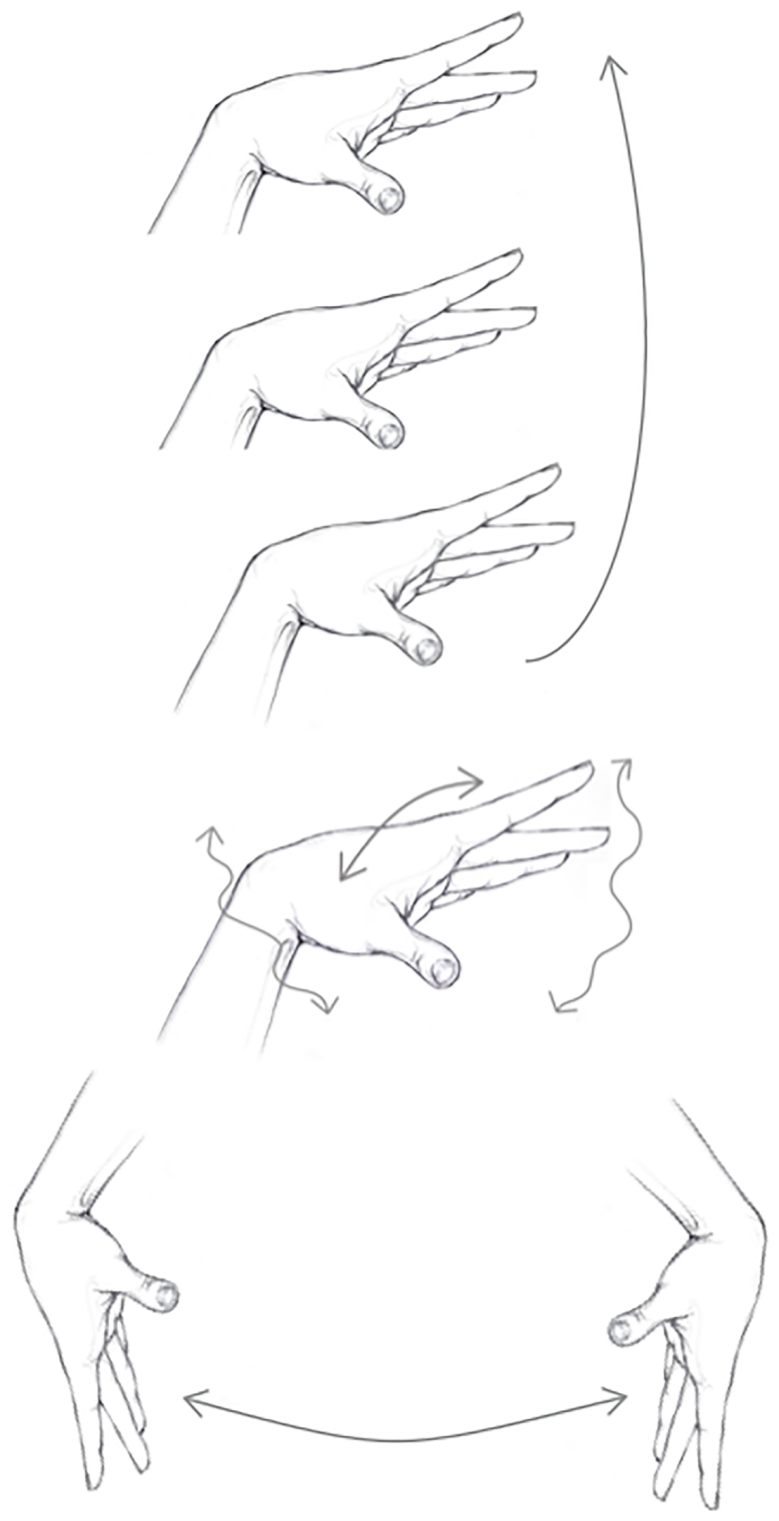
Top panel: most participants used the vertical dimension to represent pitch (“spatial metaphor of pitch”). Middle panel: many participants also rapidly shook their arms, hands, and fingers to express random fluctuations of the sounds. Bottom panel: some participants created a gap between their hands to contrast noisy sounds from tonal sounds.

For the refrigerator, most imitators (80%) rapidly shook their arms, hands, or fingers (see the middle panel of Fig 2), sometimes combined with a horizontal gesture. All the imitators imitated the upward sweep with an upward vertical gesture (see the top panel of Fig 2), sometimes combined with a outward, leftward, rightward, or even downward movement. Interestingly, only 70% of the imitators produced a downward movement for the downward sweeps and 40% produced an *upward* movement (looking in more detail, two imitators combined upward and downward movements, and two imitators produced unambiguously upward gestures). The vertical dimension is therefore clearly shared among imitators, but the assignment of low and high pitches to low and high positions is not systematic.

The annotators also noted that many imitators produced a gesture consisting of opening the hands or having both hands getting apart, as if indicating the opening of a narrow gap (see the bottom panel of Fig 2). For example, 40% of the imitators extended their fingers and opposed hands while imitating the upward sweeps, as imitating the opening of gap; another imitator closed their fingers while imitating the downward sweep as if closing a gap.

**2.3.1.2 Simple noisy sounds.** As expected, most imitators produced noisy vocalizations to imitate the noisy referent sounds (90% for the door sound, 100% for the stationary noise, 100% for the scraping sound). Most of them (80%) also rendered the repetition of the scraping sound by spectrally modulating the vocalizations, sometimes breathing in and out.

Most of the imitators imitated the door sound with a downward pitch or spectral centroid (70%). They also produced a leftward (60%), upward, or outward rapid gesture mimicking the action of closing a door. Most participants accompanied the imitation of the stationary noise by rapidly shaking their arms, hands or fingers (80%), without following any particular direction (see the top panel of Fig 2). The imitators also produced a back and and forth gesture in synchrony with the vocalization to imitate the scraping sound.

**2.3.1.3 Combined sounds.** The imitators produced complex combinations of vocalizations and gestures for the sound of filling a receptacle. All of them produced noisy vocalizations with spectral modulations (80%), roughness (60%), and upward pitched components (60%), reproducing the evolution of the sound while the receptacle is being filled. They also used an upward (60%) or downward (30%) gesture, probably mimicking the filling of the receptacle.

Every imitator produced a tonal vocalization for the printer, imitating the tonal part of this referent sound, sometimes combined with a noisy component. Many imitators (70%) also added upward sweeps imitating the squeaks of the mechanism outputting the pages. Most of them also used a combination of upward and downward (80%), inward and outward or leftward and outward gestures, somehow mimicking the movement of the pages being printed out.

#### 2.3.2 Co-occurrences

Next, the analysis counted whenever vocal and gestural features co-occurred. The percentages reported in the next paragraphs are calculated with reference to the total number of imitations per referent sound: therefore, “100%” means that the gestural and vocal features co-occurred in 100% of the imitations of this referent sound. As before, we only report co-occurrences occurring in more than 60% of the imitations. The complete data are reported in Figs A to H in S1 Appendix.

**2.3.2.1 Simple tonal sounds.** For the refrigerator, the most common co-occurrence (80%) consisted of a tonal vocalization associated with a shaking gesture with no particular direction (60%). For the upward sweeps, 100% of the imitations associated upward vocalizations with an upward gesture. For the downward sweeps, 70% of the imitations associated downward vocalizations with a downward gesture (40% with an upward gesture; several gestures therefore combined downward and upward gestures).

**2.3.2.2 Simple noisy sounds.** For the door slam, 60% of the imitations associated noisy vocalizations with a leftward gesture. These leftward gestures correspond to the subjects pantomiming the slamming of the door with their right hand. For the stationary noises, 80% of them associated noisy vocalizations with shaky gestures, and 70% with a gesture with no particular direction. For the scraping sound however, none of the noisy vocalizations co-occurred with a shaking movement. Instead, 100% of imitations associated noisy vocalizations with a back and forth movement (left/right, in/out, or up/down). The back and forth gesture may be interpreted as pantomiming the action of smoothing an object with sandpaper.

**2.3.2.3 Combined sounds.** For the sound of filling a receptacle, 60% of the imitations associated a noisy vocalization with a shaking gesture, moving upward (60%) or sometimes downward. The upward direction could correspond to the imitators’ indicating the filling of the receptacle. For the printer, 90% of the imitations associated tonal vocalizations (a succession of upward and downward sweeps) with back and forth gestures (80% up/down). These tonal/directional aspects probably correspond to the squeaks of the mechanism.

#### 2.3.3 Self-confrontation interviews

The self-confrontation interviews complemented the previous analyses. The most common comment was that the imitators had pantomimed some aspects of the sound sources despite the instructions: sawing, water dripping, waves, etc.

Then, most of the imitators mentioned a spatial metaphor for the pitch. The most common metaphor associates the vertical direction with pitch (a low position corresponding to a low pitch and vice versa). Several imitators also mentioned that they associated upward sweeps with the sound going away from them (along the medial axis) and downward sweeps with the sound coming toward them. Two imitators imitated the sweeps with a gesture that moved in the opposite directions, and mentioned that they wanted to emphasize the onset of the sounds: they felt that the onset was moving in the opposite direction of the main movement of the pitch. Several imitators mentioned that they associated a high position with high frequencies because they perceived higher frequencies as harsher, close to their ears. Note that some imitators mentioned loudness instead of pitch. It is not clear that they actually heard crescendos and decrescendos or whether they confused the two terms. In the same idea, many imitators reported that they did a movement in the horizontal plane to represent the absence of an evolution of pitch.

Another common comment was that they pinched their fingers when they felt that the sound became more precise (or higher in pitch), and opened their hands when they felt that the sound was becoming fussier (or lower in pitch).

Several imitators also mentioned that they shook their hands or fingers to represent the randomness of the sounds.

### 2.4 Discussion

The first striking result of the observational study is that there exists several vocal and gestural regularities shared among imitators. Vocal imitations reproduce rather directly some relevant features of the referent sounds: noisy textures are imitated with noisy vocalizations, tonal sounds with tonal vocalizations, etc. What is more surprising is these vocal features co-occur quite systematically with specific gestural features. These associations seem based on metaphors shared by the imitators.

The first of such metaphor is the ubiquitous spatial metaphor of pitch (see the top panel of Fig 2). Most imitators have associated low frequencies with a low position of the hands along the vertical axis (and vice versa), and imitated pitch changes with consistent hand motions. This association is sometimes combined with a horizontal motion (higher frequencies being positioned away from the person). Consistently with this idea, we observed that referent sounds with no pitch or no pitch change were imitated without any such gesture. Note however that a few participants inverted the correspondence, by associating low pitches with a high position. In particular, although all participants used an upward gesture to imitate an upward sweep, two participants made a gesture composed of an upward gesture followed by a downward gesture to imitate a downward sweep. Two other participants made an unambiguously upward gesture to imitate a downward sweep.

A more unexpected association is that several imitators represented tonalness by creating a gap between their fingers or between their hands, and associated narrow gaps with higher frequencies and larger gaps with lower frequencies (see the bottom panel of Fig 2). For example, four imitators extended the fingers of their opposed hands, as if mimicking the opening of a gap in the horizontal direction, while imitating the upward sweeps. A similar association (high pitches are thin and low pitches are thick) has been reported in some languages (Farsi, Turkish, Zapotec). Experimental studies have further shown that Dutch speakers (the thickness-pitch association does not exist in Dutch) show sensitivity to this association (but not to the reverse association), which suggests that it is rooted on a deep cognitive association [48]. One participant indicated that she felt that the high-pitched sounds were “more precise”, but it is difficult to interpret this idea. Another potential association could be that the gestures were representing the wavelength of the sounds: wider wavelengths correspond to lower pitches and narrower wavelengths to higher pitches. However, it is unclear whether the lay participants were aware of the wavelength/frequency relationship.

Another unexpected association is that many imitators (80%) imitated noisy stationary textures and the slowly fluctuating hum of the refrigerator by rapidly shaking their hands and fingers (see the middle panel of Fig 2). The gesture appears to be metaphorically related to the random fluctuations that characterizes both these sounds. Such gestures also occurred quite often in the imitations of the complex referent sounds (filling and printer), synchronized with the noisy phases of the sounds. However, no imitator executed such a gesture to imitate the scraping sound, though this sound was extremely noisy. In this case, it appears that the gestures focused on another, probably more salient element of the referent sounds: rhythm.

Finally, the gestures used to imitate the door slam, the smoothing of a piece of wood, and in some cases the sound of filling a receptacle and the printer were clear instances of pantomime (slamming a door, smoothing a piece of wood, the motion of droplets, printing out pages) despite the instructions not to do so. It thus appears very difficult to resist the drive to pantomime the situation that produced the sound when the imitators can identify it [52].

Overall, these results suggest a tight bond between the vocalizations and gestures. For example, we observed only a few cases in which the vocalizations and the gestures represented different aspects of the sounds. In one such example, one imitator vocalized an upward sweep (representing an upward tonal motion of the referent sound) while shaking his fingers (representing the noisy characteristics of the sounds). In another example, one imitator made a noisy, spectrally modulated vocalization while moving his hands upward. These examples are however seldom. The most common case of a dissociation between the voice and the gestures occurred for the scraping sound that imitators imitated with a rhythmic noisy vocalization associated with a back and forth movement of the hands. In several cases, the vocalization and the gesture tended to desynchronize (the gesture slowing down). One possibility is that it was physically difficult to maintain a tight synchronicity between vocalizations and gestures, especially because the experimental sessions could last a couple of hours.

One important qualification of these results is that they were observed in a setting that was not strictly controlled. For example, the referent sounds were recordings of everyday events, i.e. complex sounds with several acoustic features covarying. Furthermore, the annotations were done subjectively by the coauthors. It is therefore possible that some uncontrolled factors, or even the annotators’s potential biases, drove the results. Finally, analyses were mostly qualitative and no statistical inference could be made.

To test the generality of our results, we therefore conducted an experiment in a more controlled setting, focusing on two of the main results: the spatial metaphor of pitch and the systemic association of spectrally modulated noisy vocalizations with shaking gestures.

Thus, instead of complex ecological stimuli, the experimental study used simpler synthetic referent sounds that we specifically created to isolate the factors that we assumed to influence the gesture: the random fluctuations of the sounds and the pitch direction. The experiment used abstract sounds (i.e. sounds with no identifiable mechanical source) [52]. Instead of using annotations, the experimental study analyzed the data with quantitative descriptors using wrist acceleration and the tracking of hand motion using a depth camera.

## 3 Experimental study

The observational study suggested that imitators used a number of shared metaphors to communicate the referent sounds. The goal of the second, experimental study was to confirm these metaphors in a controlled, experimental setting, using specifically designed sounds, quantitative measurements, and inferential statistics.

Based on the results of the observational study, we created stimuli by crossing three factors: morphological profiles (i.e. stationary sounds vs. upward sounds), tonalness (i.e. tonal vs. noisy stimuli), and granularity (smooth vs. granular sounds). Morphological profiles and tonalness correspond to the factors already described in the observational study. Granularity corresponds to a type of modulations that are slower than those produced by random noises, and resulting in the perception of irregular “details” affecting the surface of the sounds (see for example http://www.auralsonology.com/the-signs/chapter-4-spectromorphology/, last retrieved on April 4, 2017). Granular sounds were synthesized by concatenating multiple grains (short sound samples of typically 50 ms), each of them possessing random phase variations (pitch, duration, and/or onset time) within fixed intervals. The resulting sounds thus presented random, but somewhat stable fluctuations. We used synthetic, meaningless sounds to prevent participants from imagining and pantomiming the source of the sounds.

Based on the results of the observational study, we expected vocalizations to match both the tonalness and the morphological profiles of the sounds. We expected the gestures to move along a main vertical direction for the morphological profiles of pitch, and the speed of the gestures to express the modulations of the referent sounds. The goal of the study was also to assess whether the combination of profiles and tonalness would affect these vocal and gestural features. In addition, the study observed whether vocal and gestural features would reflect two types of random fluctuations (noisiness and granularity).

We used quantitative measurements based on the vocal signals, the position and acceleration of the wrists, instead of annotations. These metrics measured the tonalness, pitch contour, and jitter (fluctuation of pitch) of the vocalizations, and the speed, spectral content, and direction of the gestures. The metrics were selected and designed to measure the vocal and gestural phenomena expected from the results of the observational study.

### 3.1 Material: Synthetic textures

**Fig 3:**
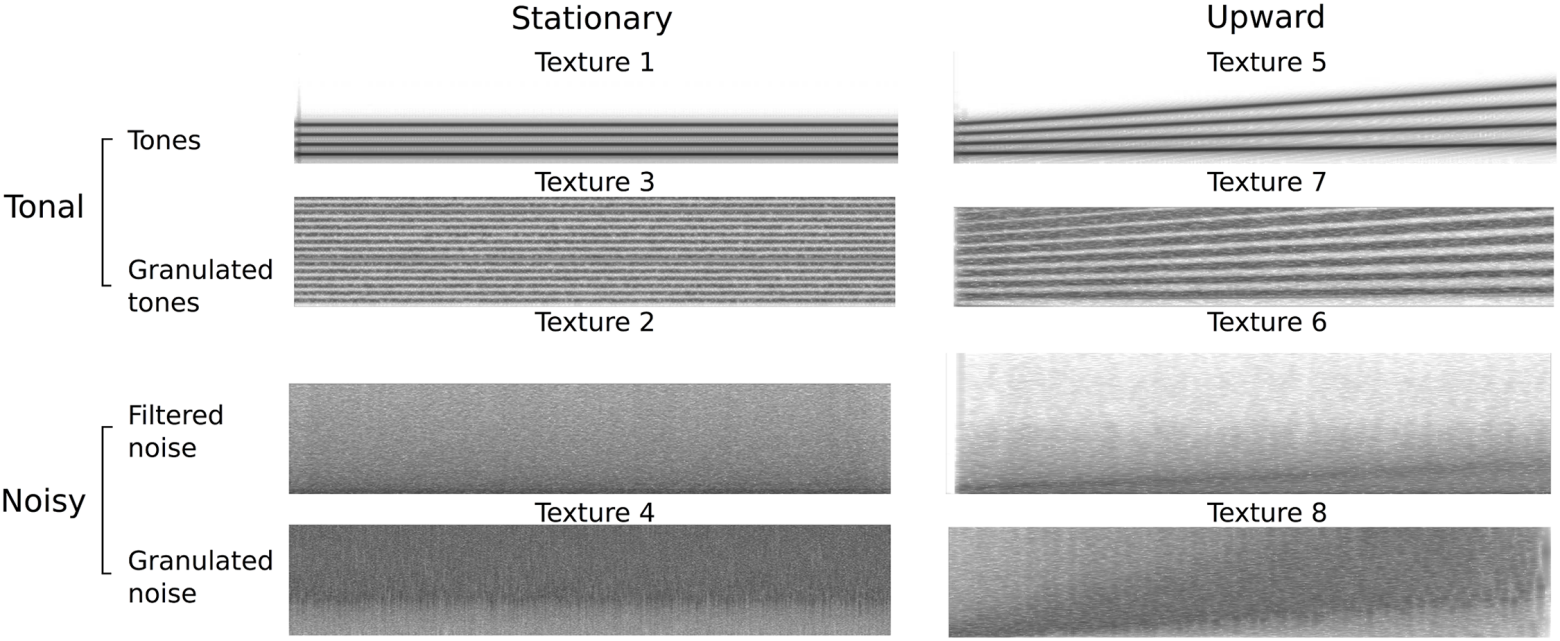
Spectrograms of the eight referent textures used in the experimental study. The left panel represents the four stationary textures (1-4), the right panel represents the four upward textures (5-8). Odd-numbered textures (1, 3, 5, 7) are tonal, even-numbered textures (2, 4, 6, 8) are noisy. Among tonal textures, textures 1 and 5 are (pitched) tonal (series of pure tones), whereas textures 3 and 7 are (tonal) granulated textures (resulting from granular synthesis based on a database of pure tones). Among noisy textures, textures 2 and 4 are based on filtered white noise, whereas textures 6 and 8) are made of granulated noises.

We created eight referent sounds (called here “synthetic textures” to distinguish them for the sound recordings used in the observational study). We first created four textures with a stationary profile. Texture 1 is a stationary, tonal, smooth texture. It consists of a 5-s, 220-Hz tone with three harmonic partials. Texture 2 is a stationary, noisy smooth texture. It consists of a 5-s, narrow-band noise (center frequency: 250 Hz, bandwidth 300 Hz). Texture 3 is a stationary, tonal, granulated texture. It consists of a 8-s, 165-Hz sawtooth passed trough a granular synthesizer (Ircam’s “Smooth overlap granular synthesis”: http://ismm.ircam.fr/maxmsp-externals/, last retrieved on April 4, 2017), with 60-ms grains and onset variation of 0.025 ms. Texture 4 is a stationary, noisy, granulated texture. It was created by passing a 7-s sawtooth through the granular synthesizer multiple times (7-ms grains with 200-ms onset variations) until it completely looses the tonal structure. We also synthesized four textures with an upward profile by increasing over time the pitch of the tonal sounds or the center frequency of a bandpass filter applied to a broadband noise. Texture 5 is an upward, tonal, smooth texture. It consists of a 4-harmonic complex (i.e. similar to Texture 1) with a fundamental frequency linearly increasing from 220 to 440 Hz in 2 s. Texture 6 is an upward, noisy, smooth texture. It consists of filtering Texture 2 with a narrow-band filter whose frequency linearly increases from 1000 to 5000 Hz in 2 s. Texture 7 is an upward, pitched, granulated texture. It was created by passing a sawtooth (hence similar to Texture 3) with a fundamental frequency linearly increasing from 330 Hz to 500 Hz in 2 s through the granular synthesizer (grain size 20 ms, onset variation 0.7 ms). Texture 8 is an upward, noisy, granulated texture. It was created by passing a sawtooth filtered with a narrow-band filter with a center frequency linearly increasing from 100 Hz to 1500 Hz (in 2 s) through the granular synthesizer multiple times (7-ms grains with 200-ms onset variations) until it completely looses the tonal structure (hence similar to Texture 4). Fundamental frequencies and sweep parameters were chosen regarding human vocal tract abilities [72, 73]. The spectrograms of the eight referent sounds are represented in Fig 3.

This study was approved by the Institutional Review Board of the French National Institute of Medical Research (CEEI-IRB Inserm 15-2160).

### 3.2 Method

#### 3.2.1 Participants

Eighteen persons (ten male, eight female), from 18 to 45 years old (median 25 years old), volunteered as participants. All reported normal hearing and were native speakers of French. None of them have musical, dancing, or sign language expertise.

#### 3.2.2 Procedure and experimental setup

The experiment used the same interface, experimental setup, and the same procedure as in the observational study. In addition, the right-hand position was tracked using a depth camera (Microsoft Kinect v2). The Kinect data (position of the limbs in three dimensions over time) were acquired and managed using Dale Phurrough’s Max/MSP external (https://hidale.com, last retrieved on June 14, 2014). As in the observational study, the instructions emphasized the communicative goal of the imitations. The task for the participants was to imitate the referent sounds so that somebody else could recognize them only by listening and watching the imitation. They were not allowed to use onomatopoeias. They were not allowed to pantomime the imagined sound-producing action. They had to use only their right hand (all participants were right-handed). Accordingly, only the data of the right hand were analyzed.

### 3.3 Vocal and gestural descriptors

We computed several vocal and gestural descriptors to account for the potential strategies used by the imitators to depict the referent sounds. For the sake of legibility, we report in this section the details of the computation of these descriptors, since some of them were specifically created for this study.

#### 3.3.1 Vocal descriptors

We computed several vocal descriptors: the aperiodicity, the slope of fundamental frequency for the imitations of tonal referent sounds, the slope of spectral centroid for the imitations of the noisy referent sounds, the jitter of tonal signals, and the amplitude modulation of the noisy signals.

**3.3.1.1 Aperiodicity.** Aperiodicity was computed using the YIN algorithm [74]. It is a measure of the noisiness of a signal: it measures the ratio of aperiodic power to the total power of the signal, and ranges from 0 (tonal signals) to 1 (noisy signals). Therefore, voiced vocalizations (i.e. involving the vibrations of the vocal folds or other soft tissues in the vocal apparatus) have small aperiodicity values, whereas unvoiced vocalizations (i.e. produced by turbulences in the vocal apparatus such as in whispering or expelling air through pursed lips) have larger values.

**Fig 4:**
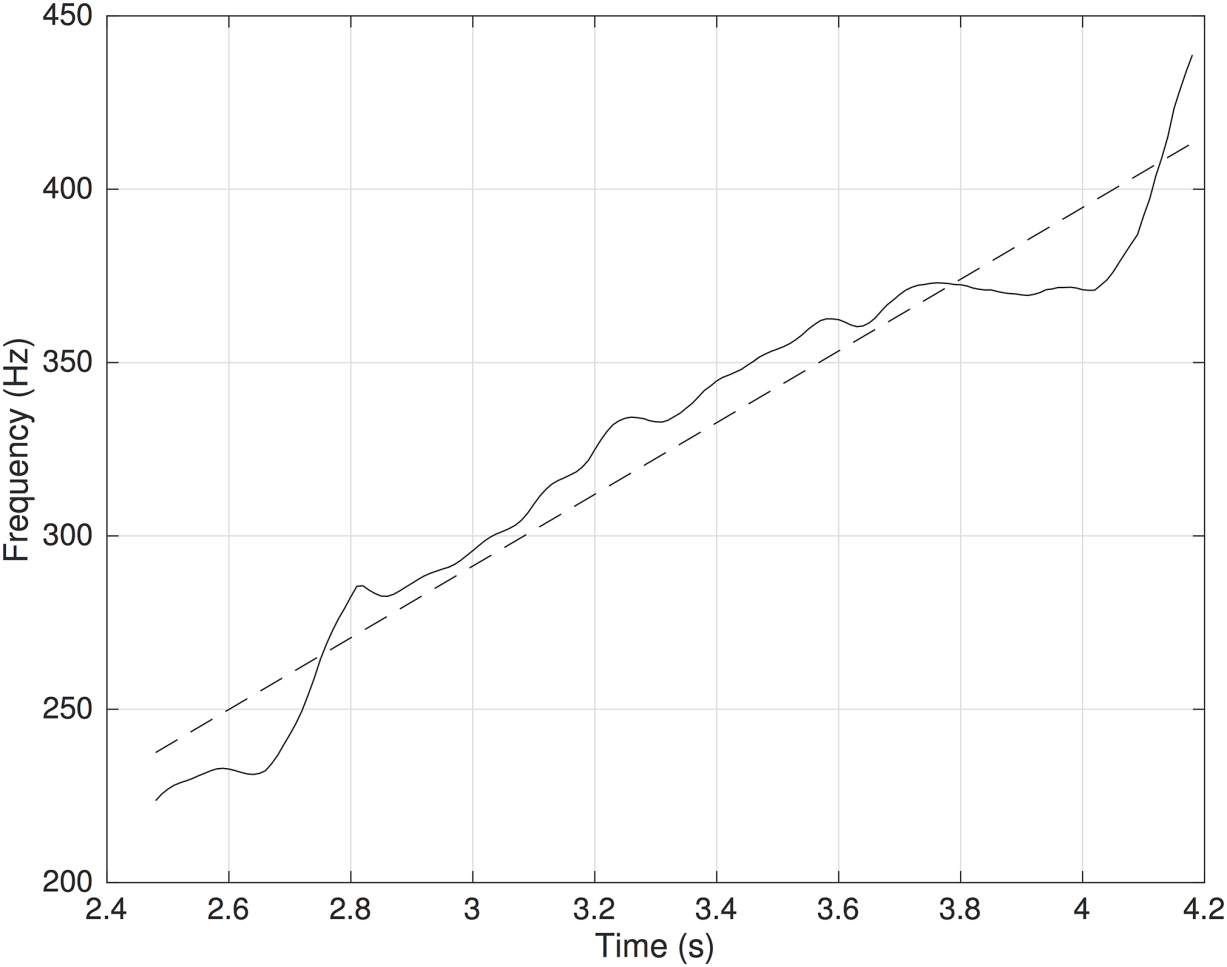
Example of regression for pitch. The slope of the regression was used as an estimate of the direction of the pitch.

**3.3.1.2 Slopes of pitch and spectral centroid.** For the voiced vocal imitations of pitched textures (1, 3, 5, 7), we computed the time-varying estimation of fundamental frequency provided by the YIN algorithm. To focus on the overall pitch increase rather than small variations of fundamental frequency, we approximated the trajectory of the pitch over time with a linear regression (fundamental frequency as a linear function of time) and used the slope of the regression to estimate the direction of the pitch of the vocalizations (see Fig 4).

For unvoiced vocal imitations of noisy textures (2, 4, 6, 8), we applied the same method and calculated the slope of the spectral centroid of the vocalization using the Timbre Toolbox [75]. The spectral centroid measures the balance of energy in a signal. Sounds with a lot of energy in higher frequencies have thus a high spectral centroid.

Both these measures indicate whether participants made a stable (slope equals zero) or an upward vocalization (slope > 0).

**3.3.1.3 Jitter.** To account for the imitations of granular tonal sounds, we also calculated the jitter with Praat [76]. We used the “local” method of Praat (pitch was first estimated between 75 and 500 Hz, and the method used a maximum period factor of 1.3 and a maximum amplitude factor of 1.6). This measures the average absolute difference between consecutive periods, divided by the average period. This local jitter is thus a measure of the local fluctuations of pitch, which imitators may use to vocalize the granularity of tonal sounds.

**3.3.1.4 Amplitude modulation depth.** To account for the imitations of granular noisy sounds, we computed the depth of amplitude modulation of the signals using the Timbre Toolbox [75]. In fact, we expected imitators would use such modulations to vocalize the granularity of tonal sounds.

#### 3.3.2 Gesture descriptors

As important characteristics of the movement appear to be related to various oscillating patterns of the hand, we chose to use a time-frequency representation for the data analysis. In particular, we opted for the Continuous Wavelet Transform (CWT) [77] that addresses some of the limitations of the Windowed Fourier Transform (WFT) for movement analysis. In fact, in Fourier analysis, the spectrum of a signal is estimated by convoluting the signal by a set of harmonic plane waves. The Windowed Fourier Transform (WFT)—or Short-term Fourier Transform—derives a time-frequency representation of signals by performing a Discrete Fourier Transform on a sliding window along the signal. The WFT therefore assumes a fixed window size, which determines the bandwidth of each frequency band in the time-frequency representation. One of the main limitations of the WFT is thus the inaccuracy resulting from the imposition of a scale or ‘response interval’ into the analysis [78]. Indeed, the WFT aliases all frequency components that do not fall within the frequency range of the window. On the contrary, instead of assuming a fixed window size, the Wavelet Transform both translates and dilates a wavelet function with short-term influence [77]. The Continuous Wavelet Transform is an alternative method that computes a time-frequency representation (called scalogram) with optimal time-frequency localization [79]. The frequency bands in the resulting time-frequency representation are logarithmically distributed, with a bandwidth that varies with the frequency, so that the number of bands per octave is constant. This provides an increased frequency resolution in low frequencies, and an increased temporal resolution in high frequencies.

For each gesture, we computed the power spectrum of CWT of the acceleration in the x, y and z axes, from the acceleration data of the IMUs. We used the Complex Morlet wavelet basis with a carrier frequency *ω*_0_ = 5*Hz*, 8 bands per octave, on the 0.1–50 hz frequency range [78]. This provides us with a time-frequency representation of the gesture data in each direction, called scalogram. In a scalogram, the spectral dimension (the wavelet spectrum) is characterized by spectral bands that are distributed in “scales” that evolve as the inverse of a frequency (a low scale therefore corresponds to a high frequency). We focused on the extraction of gesture-level descriptors from the scalogram, using the moments (centroid and spread) of the average wavelet spectrum, as described below.

**3.3.2.1 Centroid of the wavelet spectrum.** For each gestural imitation, we computed the average power spectrum in the wavelet domain by averaging the scalogram across the time axis. Taking the centroid of the wavelet power spectrum gives an approximation of the frequency of the gesture which is useful to identify rapid oscillations. A low spectral centroid in the wavelet spectrum value indicates a shaky gesture (high frequency), and high specral centroid value indicates a smooth gesture (low frequency).

**3.3.2.2 Spread of the wavelet spectrum.** We also computed the spread of the wavelet power spectrum, averaged over the duration of a gesture. This descriptor relates to the complexity of the gestures: the scalogram is more spread out when the gestures makes a complex pattern than when the gesture makes a simple sinusoidal oscillation.

**3.3.2.3 Hand vertical velocity.** We used the Kinect data to measure the distance travelled by the right hand in the vertical dimension (y-axis). Similarly to what we did with the pitch profiles, the trajectory of the right hand was first modeled by a linear model and we estimated the slope of the regression, which corresponds to the velocity of the right hand along the vertical dimension.

### 3.4 Results

We analyzed separately the vocal and gestural features of the imitations.

#### 3.4.1 Vocal strategies

**Fig 5:**
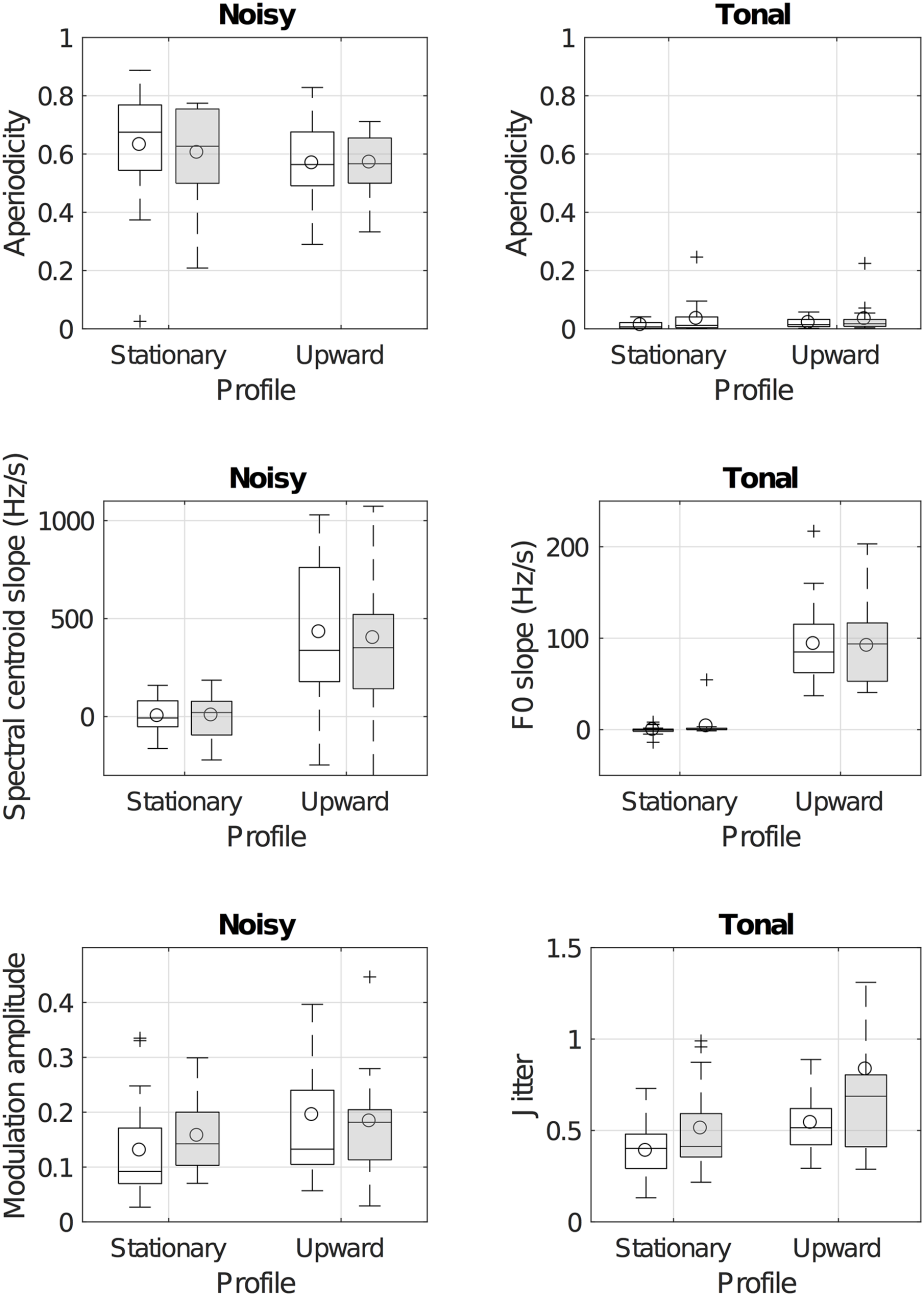
Box plots of the distributions of descriptor values for the vocal imitations of textures for the factors tonalness, granularity, and morphological profiles of the referent sounds. The boxes represent the interquartile range. Horizontal bars within the boxes represent the median of the distributions. Circles represent the mean of the distributions. The dashed vertical lines represent the ± 2.7 standard deviation range. Crosses represent outliers outside this range. Empty boxes correspond to smooth textures. Shaded boxes correspond to granular texures. The top panel represent the aperiodicity of the imitations. The middle panel represents the “spectral direction” of the vocalizations (the pitch or spectral slope). The left bottom panel represents the depth of amplitude modulations for the imitations of noisy referent sounds. The right bottom panel represents the jitter of the imitations of tonal referent sounds.

Fig 5 represents the vocal features of the vocal imitations for the tonalness, granularity, and morphological profiles of the referent sounds.

**3.4.1.1 Aperiodicity of the vocalizations.** The top panel of Fig 5 represents the aperiodicity of the imitations. Aperiodicity was submitted to a repeated-measure ANOVA with Tonalness, Granularity, and Profile as within-participant factors. With an *α* level of .01, only the effect of Tonalness was significant (F(1, 17) = 1035, p = 1.12x10^−16^, $\eta_{G}^{2}=.87$ [80]). Neither the effect of Granularity (F(1, 17) = 0, p = .90, $\eta_{G}^{2}=.00$), Profile (F(1, 17) = 1, p = 0.31, $\eta_{G}^{2}=.01$), the two-way, nor the three-way interactions were significant. As expected, imitators systematically produced tonal vocalizations for the tonal referent sounds and vice versa. This effect was not influenced by the other factors in any way.

**3.4.1.2 Spectral direction of the vocalizations.** Because imitations of tonal referent sounds were systematically tonal and imitations of noisy referent sounds were systematically noisy, we used the pitch slope for the imitations of tonal textures, and the spectral centroid slope for the imitations of noisy textures (represented in the middle panel of Fig 5).

Fundamental frequency slopes were submitted to a repeated-measure ANOVA with Granularity, and Profile as within-participant factors, for the imitations of the tonal sounds only. With an *α* level of .01, only the effect of the morphological Profiles was significant (F(1, 17) = 53.1, p = 1.26x10^−6^, $\eta_{G}^{2}=.52$). Neither the effect of Granularity (F(1, 17) = 0.4, p = 0.53, $\eta_{G}^{2}=.00$) nor the interaction between Profile and Granularity (F(1, 17) = 1.7, p = 0.21, $\eta_{G}^{2}=.02$) were significant.

Spectral centroid slopes were submitted to a repeated-measure ANOVA with Granularity, and Profile as within-participant factors, for the noisy sounds only. With an *α* level of .01, only the effect of the morphological Profiles was significant (F(1, 17) = 11.8, p = .00314, $\eta_{G}^{2}=.18$). Neither the effect of Granularity (F(1, 17) = 1.2, p = 0.28, $\eta_{G}^{2}=.01$) nor the interaction between Profile and Granularity (F(1, 17) = 2.1, p = 0.16, $\eta_{G}^{2}=.02$) were significant.

Overall, these results show that the imitators produced imitations with an upward pitch or spectral centroid, whenever the referent sounds had an upward profile, as expected. This effect was independent of the other factors. For the tonal sounds, data also suggest that participants approximately matched the speed of the pitch change (about 100 Hz/s).

**3.4.1.3 Jitter and amplitude modulation of the vocalizations.** For the tonal sounds, the jitter was submitted to an ANOVA similar to previously described. With an *α* level of .05, both the effects of Profile (F(1, 17) = 6.3, p = .0225, $\eta_{G}^{2}=.10$) and Granularity (F(1, 17) = 7.7, p = .0128, $\eta_{G}^{2}=.08$) were significant, whereas the interaction between the two factors was not significant (F(1, 17) = 1.1, p = 0.30, $\eta_{G}^{2}=.01$). Overall, for the tonal referent sounds, the pitch of the vocalizations was more fluctuant when the referent sounds were granular than when they were smooth, and when the pitch of the sounds was moving upward.

For the noisy sounds, the modulation amplitude was submitted to the ANOVA. With an *α* value of .05, only the effect of the profile was significant (F(1, 17) = 4.8, p = .0422, $\eta_{G}^{2}=.04$). The effect of Granularity (F(1, 17) = 0.1, p = 0.76, $\eta_{G}^{2}=.00$) and the interaction between the two factors (F(1, 17) = 0.6, p = 0.46, $\eta_{G}^{2}=.01$) were not statistically significant. Overall, the granularity did not seem to have an effect on the modulation of the noisy imitations.

#### 3.4.2 Gestural strategies

**Fig 6:**
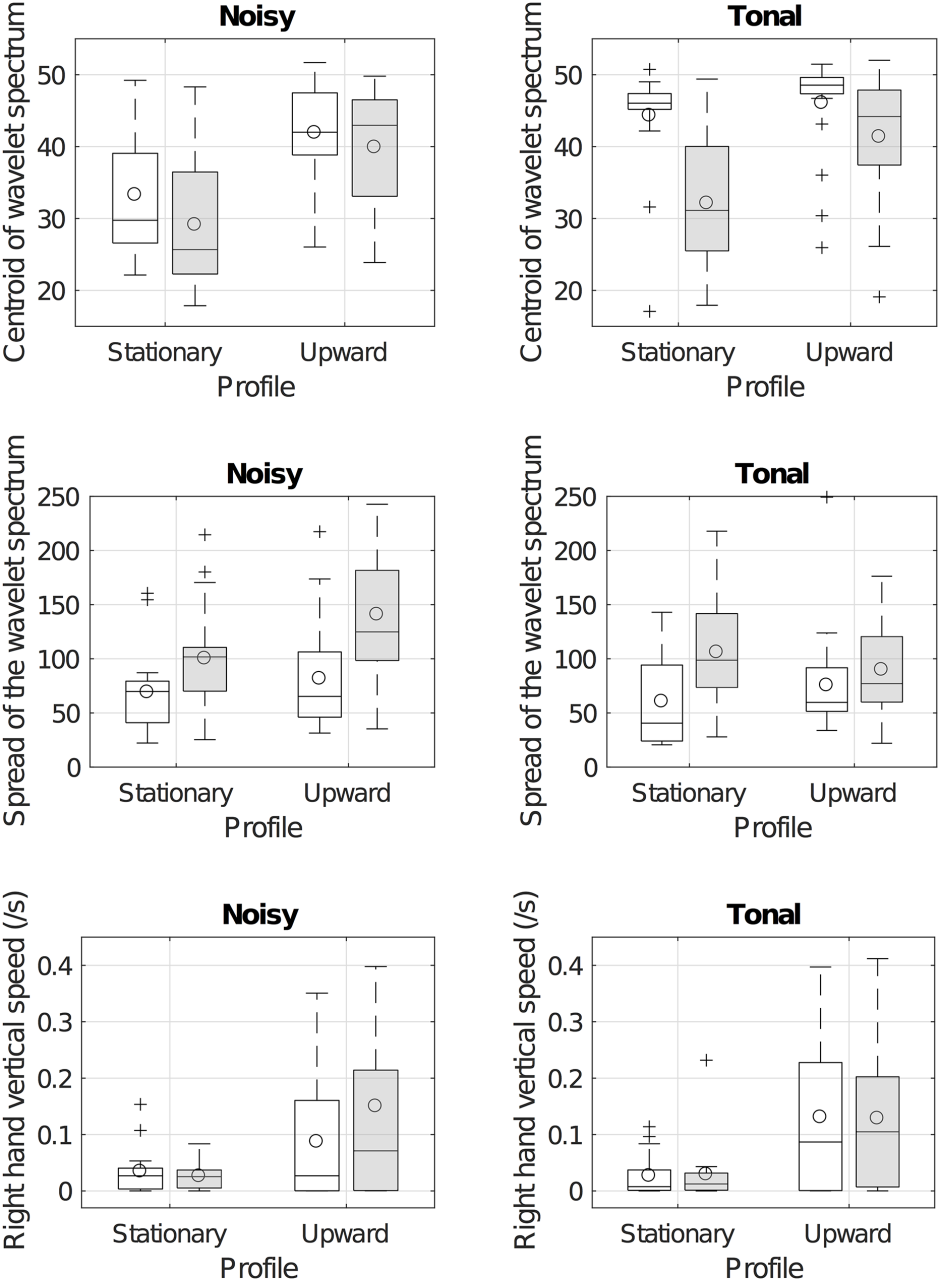
Box plots of the distributions of the gestural descriptors for the tonalness, granularity, and profile of the referent sounds. Empty boxes correspond to smooth textures, gray boxes correspond to granular textures (see the legend of Fig 5 for more detail). The top panel represents the centroid of the wavelet spectrum of the wrist acceleration, corresponding to the speed of the gestures (higher values corresponding to slower speeds). The middle panel represents the spread of the wavelet spectrum of the wrist acceleration, corresponding to the complexity of the gestures. The bottom panel represents the speed of the right hand along the vertical direction.

Fig 6 represents the gestural features of the vocal imitations for the tonalness, granularity, and morphological profiles of the referent sounds.

**3.4.2.1 Rapid fluctuations: centroid of wavelet spectrum.** The top panel of Fig 6 represents the centroid of the wavelet spectrum. The gesture centroid was submitted to a repeated-measure ANOVA with Tonalness, Granularity, and Profile as within-participant factors. With an *α* level of .01, the main effect of all three factors was significant: centroid was significantly lower (i.e. gestures were more rapid) for noisy than tonal sounds (F(1, 17) = 15.6, p = .00102, $\eta_{G}^{2}=.06$), for granular than smooth sounds (F(1, 17) = 21.7 p = .000224, $\eta_{G}^{2}=.10$), and for stationary than upward sounds (F(1, 17) = 33.2 p = 2.30x10^−5^, $\eta_{G}^{2}=.20$). The only significant interaction was between Tonalness and Granularity (F(1, 17) = 9.41 p = .007, $\eta_{G}^{2}=.04$). Post-hoc Tukey tests showed that imitations of noisy referent sounds were significantly more rapid (i.e lower centroid of the wavelet spectrum) than tonal sounds for smooth sounds (the difference is 7.6, p = .001) but not for granular sounds (the difference is 0.6, p = 0.99). Similarly, imitations of granular sounds were more rapid than smooth sounds for tonal sounds (the difference is 8.87, p = 9.05x10^−5^), but not for noisy sounds (the difference is 1.9, p = 0.77). Conversely, the interaction between tonalness and profile, between granularity and profile, and the three-way interaction were not significant (respectively (F(1, 17) = 3.2, p = .09, $\eta_{G}^{2}=.01$; F(1, 17) = 2.8, p = .14, $\eta_{G}^{2}=.03$; F(1, 17) = 2.2, p = .16, $\eta_{G}^{2}=.01$).

Overall, these results show that imitators produced more rapid gestures for both noisy and granular textures. Moreover, the effects were not additive but interacted: the gestures were more rapid for noisy and granular textures than predicted by the simple addition of the two effects. These effects did however not depend on whether the referent sounds were stationary or upward.

**3.4.2.2 Complexity of the gestures: spread of the wavelet spectrum.** The middle panel of Fig 6 represents the spread of the wavelet spectrum. The spread of the wavelet spectrum was submitted to an ANOVA similar to those previously described. With an *α* level of .05, both Tonalness (F(1, 17) = 22.9, p = .000171, $\eta_{G}^{2}=.04$) and Granularity (F(1, 17) = 18.9, p = .000435, $\eta_{G}^{2}=.14$) had a significant effect: the spread was larger (i.e. gestures were more complex) for noisy and granular sounds. The interaction between these two factors was non significant (F(1, 17) = 2.0, p = 0.17, $\eta_{G}^{2}=.01$). Profiles had no significant effect (F(1, 17) = 1.4, p = 0.25, $\eta_{G}^{2}=.02$), but interacted significantly with tonalness (F(1, 17) = 5.1, p = .0373, $\eta_{G}^{2}=.02$). The three-way interaction was also significant (F(1, 17) = 4.9, p = .0403, $\eta_{G}^{2}=.02$).

Post-hoc Tukey tests showed that the main effect of Tonalness depended on the Profile of the referent sounds. For example, the spread of the wavelet spectrum was not larger for noisy than smooth textures in the case of stationary referent sounds (p = .968, the difference is -5.45), and the difference was significant and larger in the case of upward referent sounds (p = .037, the difference is 32.3). Such an interaction was not observed for the effect of Granularity.

Overall, these analyses suggest both the tonalness and the granularity affected the complexity of the gestures: granular and noisy referent sounds were imitated with more complex gestures, although the effect of granularity seems more robust and independent than the effect of tonalness.

**3.4.2.3 Hand vertical velocity.** The bottom panel of Fig 6 represents the absolute value of the speed of the right hand in the vertical dimension. Right hand vertical speed was submitted to an ANOVA similar as those previously described (two participants were however excluded from this analysis because of missing data). With an *α* level of .01, only the Profile of the referent sounds had a significant effect (F(1, 15) = 16.8, p = .000955, $\eta_{G}^{2}=.19$): The vertical speed of the hand was significantly higher for non-stationary sounds, showing that participants clearly used the vertical dimension to indicate a pitch or spectral centroid increase. Note that about 30% of the participants produced a downward gesture for the upward profiles.

#### 3.4.3 Self-confrontation interviews

The self-confrontation interviews shed a light on the different gestures used by the participants. First most participants mentioned that they made upward gestures to express the upward motions of the sweeps (“it goes up”). Several participants also indicated that they made a horizontal gesture to illustrate the duration of the sounds. Several participants also indicated that they had used one finger or a narrow pinch to signify that the tonal sounds were made of a single note, whereas they used wide-open hands to signify that noisy sounds were more “massive” (see the bottom panel of Fig 2; note that this idea is not far from the concept of “mass” in the Schaefferian terminology [24]).

The participants used different types of description of the noisy and granular sounds. Some of them indicated that they were “fuzzy” (“flou”) or not very well defined or executed (“brouillon”) and hence made irregular rapid movements to express the absence of definition. Many of them mentioned natural events: the sea, the wind, the rain, some liquid flowing or bubbling, etc. Accordingly, they described their gestures as depicting raindrops, sea waves, bubbles, rustling leaves. Anecdotally, a few of them also imagined that the granular tonal sounds were produced by a milling machine grinding a piece of metal.

Many of them also used terms such as “oscillation”, “vibration”, “sizzle” (“grésillement”), “shiver” (“tremblement”), “crackle” (“crépitement”), “hiss” (“bruissement”), “jolts” (“soubresauts”), and indicated that their gestures were actually made of rapid small movements illustrating the complex combination of small events making the referent sounds. Interestingly, most of them indicated that they were not able to make that many rapid events with their voice and thus used their hands and fingers to illustrate the density of events.

### 3.5 Discussion

The results of the experimental study confirm that the imitators were able to vocally imitate the tonal aspect of the referent sounds. They imitated tonal sounds with a voiced vocalization, and noisy sounds with an unvoiced vocalization; they could also vocally reproduce the presence of an increase of pitch or spectral centroid. They also imitated upward profiles with an upward vocalization. All these effects were independent one from another. Granularity was less clearly conveyed: tonal granular referent sounds were imitated with a fluctuating pitch (jitter), but it was not clear whether imitators conveyed the granularity of noisy referent sounds. Informal listenings of the noisy textures suggested that the imitators were in fact somehow rendering the granularity, but that our measure was not sensitive enough to capture the acoustic features conveying this piece of information.

Regarding gestures, imitators clearly used the vertical dimension to convey the idea of pitch (i.e. the spatial metaphor of pitch). Overall, the results confirmed that participants used rapid and complex gestures to convey random fluctuations: they rapidly shook their arms, hands and fingers. Imitators’ gestures included more rapid movements to imitate noisy, granular, and upward gestures than to imitate tonal, smooth, and stationary gestures. They also produced more complex gestures for granular and noisy referent sounds than for smooth and tonal referent sounds. The analysis of the interaction between the three factors allows us to disentangle the effects of granularity and noisiness. Imitators reacted to smooth textures with slow and simple gestures (i.e. no shaking), except for stationary smooth noisy textures. In particular, smooth noisy upward textures exhibited little rapid movements of the hands or fingers. In this case, imitators privileged smooth upward gestures over the shaking gesture. The pattern of results is different for the granular textures. In this case, upward granular textures were always slow and simple, whereas stationary granular textures were more rapid and complex (i.e. rapidly shaking), even in the case of tonal granular textures. Even if a pitch is clearly perceptible for these textures, the random fluctuations created by the granular synthesis is sufficient to trigger the shaking gestures.

In a nutshell, for stationary textures, any kind of fluctuation (noisiness or granularity) resulted in rapid complex gestures, probably based on a causal metaphor of sounds (such as rustling leaves). Upward textures were systematically depicted with slow, simple, and vertical gestures based on the spatial metaphor of pitch, independently of the noisiness or the granularity of the sounds.

It is however important to note that the measures used in these analyses did not capture more subtle aspects of the gestures. For example, the gestures of some participants contained two different aspects: one slow movement (indicated by a high component in the scalogram) combined with a more rapid gesture (indicated by a low component in the scalogram). In this case, the centroid of the wavelet spectrum averaged these two components, thus resulting in a useless measure. Similarly, some participants used specific hand postures. For example, some imitators raised their forefingers to imitate harmonic sounds (judging them “precise”), whereas they waved their wide-open hands to imitate noisy sounds (judging them “large”), or even clenching their fist because they felt like a sound was “stronger” than others.

## 4 General discussion

The results of this study highlight two main metaphors consistently used in the gestural depictions of sounds: the spatial metaphor of pitch and the “rustling” metaphor (i.e. rapidly shaking of hands and fingers) of random fluctuations (see the two upper panels of Fig 2). Whereas spatial metaphors of pitch have been described in number of studies across different domains, the rustling metaphor of random fluctuation has not been reported elsewhere to the best of our knowledge.

Both metaphors consist of a correspondence between a visual (the positions and movements of the hands) and an auditory stimulus (the referent sound). These metaphors are quite systematic across participants. The results also clearly show that they are selective: They select some features of the referent sounds. For example, participants used the rustling metaphor to depict stationary textures that included some kind of fluctuation (noisiness or granularity), and the spatial metaphor to depict upward sounds. When textures combined fluctuations and an upward direction (i.e. granular or noisy upward sweeps), imitators did not combine an upward gesture with rapidly shaking their hands or fingers: in this case the gestures focused only on the direction of the sounds. This could result from physical difficulties to combine two types of gestures (although it does not seem difficult to make this combination). In turn, for stationary textures, tonal textures were depicted with slow simple gestures, and noisy or granular textures were depicted with rapid complex gestures. In this case, the gestures focused only the fluctuation of the textures. It is possible that imitators chose to focus their gestures on only one property of the referent sounds because they were rendering the other properties with their voice. But, as discussed below, a symmetrical selectivity was not observed for the vocalizations. As noted by Eitan and colleagues, the different metaphors used to represent sounds interact and are not symmetrical [38].

In contrast, vocalizations successfully combined the effects of tonalness and profiles of the referent sounds: vocalizations were noisy (unvoiced) in response to noisy textures and tonal (voiced) in response to tonal textures (irrespectively of the direction of the textures), and their pitch or spectral centroid matched the direction of the referent sounds (irrespectively of the tonalness of the referent sounds). Our measures were only able to show a modest effect of the granularity of the referent sounds, only for the tonal referent sounds: the pitch of the vocalization was more jittery in response to granular than to tonal sounds. It is possible that our measure was insensitive to the relevant vocal features for the granular noisy textures. Another possibility is that imitators did not have any strategy to add more fluctuation to their already fluctuating unvoiced vocalization. However, informal listening tests suggested that it is possible to perceive a specific modulation added to the imitations of the granular textures.

Overall, this suggests a different role for the imitative vocalizations and gestures. Whereas the vocalizations reproduce several features of the referent sounds as faithfully as vocally possible, the gestures *emphasize one* salient feature with metaphors based on auditory-visual correspondances. Admittedly, the referent sounds in the experimental study were simple, the feature values had been chosen to be easily vocalized, and our previous work had shown that imitators actually use a variety of vocal strategies (that depart from faithful reproduction) to convey more complex acoustic features less amenable to vocal imitation [22, 23]. Nevertheless, the emphasizing role of the gestures is striking. As such, it could potentially be decoded by receivers to focus their attention of some aspects of the vocalizations. Further work is needed to explore such hypotheses.

From a methodological point of view, these results were confirmed by the combination of two complementary studies: an observational study with ecologically valid and complex referent sounds, and an experimental study with simple, artificial, but controlled sounds that enabled us to use quantitive measures and inferential statistics. Instead of comparing the trajectories of sound and gesture features as in other sound tracing studies [52, 58], our analyses sought to identify different types of correspondences between auditory features of the referent sounds and visual features of the gestures. In fact, most sound tracing studies assume that participants would “trace in the air” the trajectory of some acoustic features (i.e. its temporal evolution), which is in fact based on the practice of drawing parameter curves in audio software tools (thus another specialized metaphor). Initial observations and discussions with non-expert participants suggested that such a concept was foreign to them, as also observed by Caramiaux and colleagues [52]. Whereas the spatial metaphor of pitch could be considered as some kind of parameter tracing (the position of the hands follows the evolution of pitch in time), the rustling metaphor appears to be of a more categorical nature: we were not able to observe more rapid and complex gestures when we combined noisiness and granularity. Here again, further work is needed to inquire the categorical nature of such behavior.

The origin of the metaphors is somewhat puzzling. In principle, they could be based on a pure convention (e.g. as in conducting [81]) or mediated by some language convention), resulting from the statistics of usual auditory scenes [47], based on some re-enactment or pantomiming of the physical production of the sounds, on a correspondence between auditory and visual features, or on a another type of conceptual metaphor shared within a population. Despite its ubiquity in the literature, there is no clear answer as to the origin of the spatial metaphor of pitch. Many languages use spatial analogies to describe pitch (e.g. high/low in English, French, Italian, German), and this association is strong enough to interfere with gesture execution. But Dolscheid and colleagues have shown that these terms are not purely conventional (for example they cannot be reversed) and therefore must rely on more deeply engrained associations [48]. Lakoff and Johnson have argued that most Western concepts are organized in terms of spatialization metapors [36]: happy is up and sad is down, health is up and sickness is down, good is up and bad is down, rational is up and emotional is down. Such metaphors are rooted in strong cultural and physical experiences. For example, when you pile up physical objects, level increases. Similarly, the spatial metaphor of pitch could be based on cultural or physical experience. For example, the Western musical notation system also uses such a spatial representation of pitch, and pitch models in music psychology also represent pitch height along the vertical dimension [82]. Another common interpretation is that this metaphor relies on the physical production of sounds by musical instruments. Low notes are mapped to leftmost positions on a piano keyboard [44] and on the neck of guitars and luths, but the are mapped to the rightmost keyholes on flutes. They are mapped to the farthest positions of the fingers on the neck of a violin and of the slide of a trombone, to the highest positions on upright basses and violoncellos, but to the lowest keyholes on woodwind instruments (the mapping refer here to the position of the fingers or the hands to produce a given note, from the point of view of the player). So if listeners represent pitch with a spatial metaphor based on the physical production of sounds on musical instruments, they can potentially use different mappings. The most common mapping of pitch to spatial position seems to be that lower pitches are localized on a lower, more left, and closer position with respect to the listener than higher pitches that are localized on a higher, more right, and farther position. But note that about 40% of the participants in the observational study and 30% of in the experimental study used a reverse mapping (lowest pitches localized on a highest position). One possibility is that participants were not able to hear correctly the pitch direction (i.e. confusing high and low pitches). Self-confrontations interviews showed however that most of them voluntarily associated low pitches with higher positions and vice versa. Whereas the spatial representation of pitch is overwhelming, the precise mapping between pitch and spatial position varies a lot across subjects.

The origin of the rapidly shaking gestures is even more intriguing. The self-confrontation interviews suggested a few potential origins. First, several participants indicated that their rapidly shaking gestures illustrated the “fuzziness” of the noisy sounds, which reminds of synesthesia-like mappings found for example in the taketa/maluma effect [83, 84]. Just as people systematically associate certain phonetic features (“maluma”) to rounded visual shapes and some other (“takete”) to spiky, jagged shapes, they may have a systematic bias to associate random fluctuations in sounds to visual fuzziness, gesturally rendered by shaking their hands. But the most common interpretation was either causal or acoustical. In the causal interpretation, the participants mentioned and pantomimed natural events such as bubbles, raindrops, or rustling leaves. As such, participants were embodying the physical actions causing the sounds. In the acoustical interpretation, participants mentioned that their rapidly shaking gestures were illustrating the density of small events composing the referent sounds. As such, the shaking metaphor could be seen as an extension of spatial metaphors, wherein small spatial trajectories illustrate the fluctuations of pitch or loudness (which is a rather accurate definition of a noise or a granular sound). Note finally that some examples of the shaking gesture look similar to the “applause” sign in American and French sign languages. Though none of the imitators was a signer, the applause sign may have originated from a similar metaphor, depicting the complex acoustic pattern of individual claps composing a crowd applause.

It should be noted however, that our measurements did not capture more subtle effects. For example, in the latter acoustical interpretation, many participants indicated that they used gestures to indicate density of events because they could not do it with their voice. Some metaphors also seem to have been used by only a few participants (e.g. indicating the number of notes or the “mass” of the sounds as in the lower panel of Fig 2), which cannot be highlighted by our analyses, who instead focused on shared behaviors.

The shared use of such metaphors across participants nevertheless offers a number of interesting questions. A first question is whether receivers can actually decode the gestures as they do with gestures accompanying speech [14]. For example, the idea that gestures actually emphasize one salient aspect of the vocalizations remains to be tested. Another question is whether these gestures can be combined with a syntax and thus take a linguistic form, as observed in spontaneous sign languages [85]? Goldin-Meadow has suggested that gestures take a linguistic form only when they are used without speech, such as in sign language [14]. In our initial observations [1], depicting gestures always accompanied speech or non-speech vocalizations. It remains to be studied whether spontaneous depicting gestures can communicate sensory experience by themselves, or whether they need to be combined with non-speech vocalizations. For example, some theories argue that gestures are well suited to communicate sensory experience and describe tool manipulation without vocalization, and advance that gestures could have been precursors to the apparition of vocal language in humans [15, 16, 86–88].

In addition to human-human interactions, these results also offer perspectives for the design of new human-computer interactions. In recent years, sensing technologies have enabled humans to interact with computers with gestures, far beyond touch screens [89, 90] and point-and-click metaphors [91], with applications ranging from entertainment to health research [92]. Specifically, several interactive music systems have been developed based on novel metaphorical interactions between human motion, objects, and sounds [93]. For example, we have proposed to use non-speech vocalizations and gestural metaphors for the intuitive and expressive control of sound synthesis [94]. Other possible applications include performing arts and music (where artists could explore their own gestural metaphors in relation to sound) as well as education (where non-expert users could communicate auditory experiences to a sound design tool using gestural metaphors).

## Supporting information

S1 Appendix Analysis grid, Annotations, and Co-occurrence matrices. (PDF)

## References

[1] Lemaitre G, Susini P, Rocchesso D, Lambourg C, Boussard P. Non-Verbal Imitations as a Sketching Tool for Sound Design In: Aramaki M, Derrien O, Kronland-Martinet R, Ystad S, editors. Sound, Music, and Motion. Lecture Notes in Computer Sciences. Berlin, Heidelberg, Germany: Springer; 2014 p. 558–574.

[2] Wright P. Linguistic description of auditory signals. Journal of applied psychology. 1971;55(3):244–250. 10.1037/h0031025

[3] Faure A. Des sons aux mots: comment parle-t-on du timbre musical? [Unpublished Doctoral Dissertation]. École de Hautes Études en Sciences Sociales. Paris, France; 2000.

[4] Porcello T. Speaking of sound: language and the professionalization of sound-recording engineers. Social Studies of Science. 2004;34(5):733–758. 10.1177/0306312704047328

[5] Heyes C. Automatic imitation. Psychological bulletin. 2011;137(3):463–483. 10.1037/a0022288 21280938

[6] Meltzoff AN, Prinz W. The imitative mind Development, evolution and brain bases. Cambridge, UK: Cambridge University Press; 2002 364 pages.

[7] Mercado E III, Mantell JT, Pfordresher PQ. Imitating sounds: A cognitive approach to understanding vocal imitation. Comparative Cognition & Behavior Reviews. 2014;9.

[8] Chartrand TL, Bargh JA. The chameleon effect: the perception-behavior link and social interaction. Journal of Personality and Social Psychology. 1999;76(6):893–910. 10.1037/0022-3514.76.6.893 10402679

[9] Lemaitre G, Rocchesso D. On the effectiveness of vocal imitation and verbal descriptions of sounds. Journal of the Acoustical Society of America. 2014;135(2):862–873. 10.1121/1.4861245 25234894

[10] Lemaitre G, Houix O, Voisin F, Misdariis N, Susini P. Vocal Imitations of Non-Vocal Sounds. PloS one. 2016;11(12):e0168167 10.1371/journal.pone.0168167 27992480 PMC5161510

[11] Ekman P, Friesen WV. The repertoire of nonverbal behavior: Categories, origins, usage, and coding. semiotica. 1969;1(1):49–98. 10.1515/semi.1969.1.1.49

[12] Kendon A. Gesture: Visible action as utterance. Cambridge University Press; 2004.

[13] McNeill D. Gesture and thought. University of Chicago Press; 2005.

[14] Goldin-Meadow S. The role of gesture in communication and thinking. Trends in cognitive sciences. 1999;3(11):419–429. 10.1016/S1364-6613(99)01397-2 10529797

[15] Kelly SD, Iverson JM, Terranova J, Niego J, Hopkins M, Goldsmith L. Putting language back in the body: speech and gesture on three time frames. Developmental neuropsychology. 2002;22(1):323–349. 10.1207/S15326942dn2201_1 12405508

[16] Fay N, Arbib M, Garrod S. How to bootstrap a human communication system. Cognitive science. 2013;37(7):1356–1367. 10.1111/cogs.12048 23763661

[17] Valenzeno L, Alibali MW, Klatzky R. Teachers’ gestures facilitate students’ learning: A lesson in symmetry. Contemporary Educational Psychology. 2003;28(2):187–204. 10.1016/S0361-476X(02)00007-3

[18] Krauss RM, Dushay RA, Chen Y, Rauscher F. The communicative value of conversational hand gesture. Journal of Experimental Social Psychology. 1995;31(6):533–552. 10.1006/jesp.1995.1024

[19] Krauss RM. Why do we gesture when we speak? Current Directions in Psychological Science. 1998;7(2):54–54. 10.1111/1467-8721.ep13175642

[20] Goldin-Meadow S, Singer MA. From children’s hands to adults’ ears: gesture’s role in the learning process. Developmental psychology. 2003;39(3):509 10.1037/0012-1649.39.3.509 12760519

[21] Clark HH. Depicting as a method of communication. Psychological review. 2016;123(3):324–347. 10.1037/rev0000026 26855255

[22] Lemaitre G, Jabbari A, Misdariis N, Houix O, Susini P. Vocal imitations of basic auditory features. The Journal of the Acoustical Society of America. 2016;139(1):290–300. 10.1121/1.4939738 26827025

[23] Mehrabi A, Dixon S, Sandler MB. Vocal imitation of synthesised sounds varying in pitch, loudness and spectral centroid. The Journal of the Acoustical Society of America. 2017;141(2):783–796. 10.1121/1.4974825 28253682

[24] Schaeffer P. Traité des objets musicaux. Paris, France: Seuil; 1966.

[25] Gaver WW. How do we hear in the world? Explorations in ecological acoustics. Ecological Psychology. 1993;5(4):285–313. 10.1207/s15326969eco0504_2

[26] Lemaitre G, Houix O, Misdariis N, Susini P. Listener expertise and sound identification influence the categorization of environmental sounds. Journal of Experimental Psychology: Applied. 2010;16(1):16–32. 20350041 10.1037/a0018762

[27] Caramiaux B, Françoise J, Schnell N, Bevilacqua F. Mapping through listening. Computer Music Journal. 2014;38(3):34–48. 10.1162/COMJ_a_00255

[28] Vanderveer NJ. Ecological acoustics: human perception of environmental sounds [Unpublished doctoral dissertation]. Cornell University; 1979.

[29] Handel S. 8: Identification of Speakers, Instruments, and Environmental Events In: Listening: an introduction to the perception of auditory events. Cambridge, MA: The MIT Press; 1989 p. 219–274.

[30] Giordano BL, McAdams S. Material identification of real impact sounds: Effect of size variation in steel, glass, wood and plexiglass plates. Journal of the Acoustical Society of America. 2006 2;119(2):1171–1881. 10.1121/1.2149839 16521778

[31] Grassi M, Pastore M, Lemaitre G. Looking at the world with your ears: how do we get the size of an object from its sound? Acta Psychologica. 2013;143:96–104. 10.1016/j.actpsy.2013.02.005 23542810

[32] Thoret E, Aramaki M, Kronland-Martinet R, Velay JL, Ystad S. From sound to shape: auditory perception of drawing. Journal of Experimental Psychology: Human Perception and Performance. 2014;. 24446717 10.1037/a0035441

[33] Lemaitre G, Heller LM. Auditory perception of material is fragile, while action is strikingly robust. Journal of the Acoustical Society of America. 2012;131(2):1337–1348. 10.1121/1.3675946 22352507

[34] Lemaitre G, Pyles JR, Halpern AR, Navolio N, Lehet M, Heller LM. Who’s that knocking at my door? Neural bases of sound source identification. Cerebral cortex. In press;. 10.1093/cercor/bhw397 28052922

[35] Lemaitre G, Heller LM, Navolio N, Zúñiga-Peñaranda N. Priming Gestures with Sounds. PloS one. 2015;10(11):e0141791 10.1371/journal.pone.0141791 26544884 PMC4636392

[36] Lakoff G, Johnson M. Metaphors we live by. Chicago, IL: University of Chicago press; 1980 Edition 2003.

[37] Spence C. Crossmodal correspondences: A tutorial review. Attention, Perception, & Psychophysics. 2011;73(4):971–995. 10.3758/s13414-010-0073-7 21264748

[38] Eitan Z, Granot RY. How music moves: musical parameters and listeners’ images of motion. Music Perception: An Interdisciplinary Journal. 2006;23(3):221–248. 10.1525/mp.2006.23.3.221

[39] Chang S, Cho YS. Polarity correspondence effect between loudness and lateralized response set. Frontiers in psychology. 2015;6 10.3389/fpsyg.2015.00683 PMC4440908 26052305

[40] Walker R. The effects of culture, environment, age, and musical training on choices of visual metaphors for sound. Attention, Perception, & Psychophysics. 1987;42(5):491–502. 10.3758/BF03209757 2447557

[41] Melara RD, Marks LE. Processes underlying dimensional interactions: Correspondences between linguistic and nonlinguistic dimensions. Memory & Cognition. 1990;18(5):477–495. 10.3758/BF03198481 2233261

[42] Rusconi E, Kwan B, Giordano BL, Umilta C, Butterworth B. Spatial representation of pitch height: the SMARC effect. Cognition. 2006;99(2):113–129. 10.1016/j.cognition.2005.01.004 15925355

[43] Pirhonen A. Semantics of Sounds and Images-Can They Be Paralleled? In: Proceedings of the 13^th^ International Conference on Auditory Display, Montreal, Canada. Georgia Institute of Technology; 2007. p. 319–325.

[44] Lidji P, Kolinsky R, Lochy A, Morais J. Spatial associations for musical stimuli: a piano in the head? Journal of Experimental Psychology: human perception and performance. 2007;33:1189–1207. 17924817 10.1037/0096-1523.33.5.1189

[45] Lu X, Sun Y, Thompson WF. An investigation of spatial representation of pitch in individuals with congenital amusia. The Quarterly Journal of Experimental Psychology. 2016;p. 1–11. 10.1080/17470218.2016.1213870 27426027

[46] Pitteri M, Marchetti M, Priftis K, Grassi M. Naturally together: pitch-height and brightness as coupled factors for eliciting the SMARC effect in non-musicians. Psychological research. 2017;81(1):243–254. 10.1007/s00426-015-0713-6 26424464

[47] Parise CV, Knorre K, Ernst MO. Natural auditory scene statistics shapes human spatial hearing. Proceedings of the National Academy of Sciences. 2014;111(16):6104–6108. 10.1073/pnas.1322705111 PMC4000839 24711409

[48] Dolscheid S, Shayan S, Majid A, Casasanto D. The thickness of musical pitch: Psychophysical evidence for linguistic relativity. Psychological Science. 2013;24(5):613–621. 10.1177/0956797612457374 23538914

[49] Walker P, Bremner JG, Mason U, Spring J, Mattock K, Slater A, et al Preverbal Infants’ Sensitivity to Synaesthetic Cross-Modality Correspondences. Psychological Science. 2010;21(1):21–25. 10.1177/0956797609354734 20424017

[50] Lewkowicz DJ, Minar NJ. Infants Are Not Sensitive to Synesthetic Cross-Modality Correspondences A Comment on Walker et al. (2010). Psychological science. 2014;25(3):832–834. 10.1177/0956797613516011 24463555 PMC3954909

[51] Nava E, Grassi M, Turati C. Audio-visual, visuo-tactile and audio-tactile correspondences in preschoolers. Multisensory Research. 2016;29(1-3):93–111. 10.1163/22134808-00002493 27311292

[52] Caramiaux B, Bevilacqua F, Bianco T, Schnell N, Houix O, Susini P. The role of sound perception in gestural sound description. ACM Transactions on Applied Perception. 2014;11(1). 10.1145/2536811

[53] Godøy RI, Haga E, Jensenius AR. Playing “air instruments”: mimicry of sound-producing gestures by novices and experts In: International Gesture Workshop. Springer; 2005 p. 256–267.

[54] Leman M. Embodied Music Cognition and mediation technology. Cambridge, Massachusetts London, England: The MIT Press; 2008.

[55] Godøy RI, Jensenius AR, Nymoen K. Chunking in Music by Coarticulation. Acta Acustica united with Acustica. 2010;96(4).

[56] Maes PJ, Leman M, Palmer C, Wanderley MM. Action-based effects on music perception. Frontiers in Psychology. 2014;4 Article 1008. 10.3389/fpsyg.2013.01008 24454299 PMC3879531

[57] Godøy RI, Haga E, Jensenius AR. Exploring music-related gestures by sound-tracing: A preliminary study. In: Proceedings of the 2^nd^ ConGAS International Symposium on Gesture Interfaces for Multimedia Systems, Leeds, UK; 2006. p. 7 pages.

[58] Leman M, Desmet F, Styns F, Noorden LV, Moelants D. Sharing Musical Expression Through Embodied Listening: A Case Study Based on Chinese Guqin Music. Music Perception. 2009;26(3):263–278. 10.1525/mp.2009.26.3.263

[59] Nymoen K, Caramiaux B, Kozak M, Torresen J. Analyzing sound tracings: a multimodal approach to music information retrieval. In: Proceedings of the 1st international ACM workshop on Music information retrieval with user-centered and multimodal strategies. ACM; 2011. p. 39–44.

[60] Nymoen K, Godøy R, Jensenius AR, Torresen J. Analyzing Correspondence Between Sound Objects and Body Motion. ACM Transaction on Applied Perception. 2013;10(2):9:1–9:22.

[61] Caramiaux B, Bevilacqua F, Schnell N. Towards a gesture-sound cross-modal analysis In: International Gesture Workshop. Springer; 2009 p. 158–170.

[62] Caramiaux B, Bevilacqua F, Schnell N. Analyzing gesture and sound similarities with a HMM-based divergence measure. In: Proceedings of the Sound and Music Conference (SMC 2010). Barcelona, Spain; 2010.

[63] Houix O, Lemaitre G, Voisin F, Bevilacqua F, Susini P. A database of vocal and gestural imitations of sounds. Manuscript submitted for publication. 2016;.

[64] Houix O, Lemaitre G, Misdariis N, Susini P, Urdapilleta I. A lexical analysis of environmental sound categories. Journal of Experimental Psychology: Applied. 2012;18(1):52–80. 22122114 10.1037/a0026240

[65] Lemaitre G, Heller LM. Evidence for a basic level in a taxonomy of everyday action sounds. Experimental Brain Research. 2013;226(2):253–264. 10.1007/s00221-013-3430-7 23411674

[66] Zwicker E, Fastl H. Psychoacoustics Facts and Models. Berlin, Heidelberg, Gemany: Springer Verlag; 1990 463 pages.

[67] Hansen H, Verhey JL, Weber R. The magnitude of tonal content. A review. Acta Acustica united with Acustica. 2011;97(3):355–363. 10.3813/AAA.918416

[68] Peeters G, Deruty E. Sound indexing using morphological description. IEEE Transactions on audio, speech, and language processing. 2010;18(3):675—687. 10.1109/TASL.2009.2038809

[69] Rasamimanana N, Bevilacqua F, Schnell N, Guédy F, Maestracci EC, Zamborlin B, et al. Modular Musical Objects Towards Embodied Control Of Digital Music. In: Proceedings of Tangible Embedded and Embodied Interaction Conference (TEI), Funchal, Portugal. New York City, NY: ACM; 2011.

[70] Augoyard JF. L’entretien sur écoute réactivée In: Grosjean M, Thibaud JP, editors. L’espace urbain en méthodes. Marseille, France: Parenthèses; 2001 p. 127–152.

[71] Fleiss JL. Measuring nominal scale agreement among many raters. Psychological bulletin. 1971;76(5):378–382. 10.1037/h0031619

[72] Sundberg J. The science of the singing voice. DeKalb, IL: Northern Illinois University Press; 1989.

[73] Ladefoged P. Vowels and consonants: an introduction to the sounds of language. Oxford, UK: Blackwell; 2001 215 pages.

[74] de Cheveigné A, Kawahara H. YIN, a fundamental frequency estimator for speech and music. Journal of the Acoustical Society of America. 2002;111(4):1917–1930. 10.1121/1.1458024 12002874

[75] Peeters G, Giordano BL, Susini P, Misdariis N, McAdams S. The timbre toolbox: Extracting audio descriptors from musical signals. Journal of the Acoustical Society of America. 2011;130(5):2902 10.1121/1.3642604 22087919

[76] Boersma P, Weenink D. Praat: doing phonetics by computer (Version 5.1.05); 2009. Computer program. Retrieved May 1, 2009, from http://www.praat.org/

[77] Mallat S. A Wavelet Tour of Signal Processing, Third Edition: The Sparse Way. 3rd ed Academic Press; 2008.

[78] Torrence C, Compo GP. A practical guide to wavelet analysis. Bulletin of the American Meteorological society. 1998;79(1):61–78. 10.1175/1520-0477(1998)079<0061:APGTWA>2.0.CO;2

[79] Addison PS. Wavelet transforms and the ECG: a review. Physiological Measurement. 2005;26(5):R155 10.1088/0967-3334/26/5/R01 16088052

[80] Olejnik S, Algina J. Generalized eta and omega squared statistics: measures of effect size for some common research designs. Psychological methods. 2003;8(4):434 10.1037/1082-989X.8.4.434 14664681

[81] Johanssen G, Nakra TM. Conductors’s gestures and their mapping to sound synthesis In: Godøy RI, Leman M, editors. Musical gestures. Sound, Movement, and meaning. Routledge, New York City, NY; 2010 p. 264–298.

[82] Shepard RN. Circularity in judgments of relative pitch. Journal of the Acoustical Society of America. 1964;36(12):2346–2353. 10.1121/1.1919362

[83] Köhler W. Gestalt psychology. New York, NY: Liveright Publishing Corporation; 1945.

[84] Ramachandran VS, Hubbard EM. Synaesthesia–a window into perception, thought and language. Journal of consciousness studies. 2001;8(12):3–34.

[85] Schouwstra M, Smith K, Kirby S. From natural order to convention in silent gesture. In: The Evolution of Language: Proceedings of the 11th International Conference (EVOLANG XI). EVOLANG XI, New Orleans, United States. vol. 20; 2016. p. 24.

[86] Corballis MC. From hand to mouth: the origins of language. Princeton, NJ: Princeton University Press; 2002 272 pages.

[87] Corballis MC. Language as gesture. Human Movement Science. 2009;28(5):556–565. 10.1016/j.humov.2009.07.003 19665811

[88] Aboitiz F. Gestures, vocalizations, and memory in language origins. Front Evol Neurosci. 2012;4(2). 10.3389/fnevo.2012.00002 22347184 PMC3269654

[89] Pirhonen A, Brewster S, Holguin C. Gestural and audio metaphors as a means of control for mobile devices. In: Proceedings of the SIGCHI conference on Human factors in computing systems. ACM; 2002. p. 291–298.

[90] Wessel D, Wright M, Schott J. Intimate musical control of computers with a variety of controllers and gesture mapping metaphors. In: Proceedings of the 2002 conference on New interfaces for musical expression. National University of Singapore; 2002. p. 1–3.

[91] Francese R, Passero I, Tortora G. Wiimote and Kinect: gestural user interfaces add a natural third dimension to HCI. In: Proceedings of the International Working Conference on Advanced Visual Interfaces. ACM; 2012. p. 116–123.

[92] Boyer EO, Pyanet Q, Hanneton S, Bevilacqua F. Guiding Motion using a Sound Target. In: 10th International Symposium on Computer Music Multidisciplinary Research (CMMR) Sound, Music and Motion; 2013. p. 176–189.

[93] Schnell N, Bevilacqua F. Engaging with Recorded Sound Materials Through Metaphorical Actions. Contemporary Music Review. 2016;35(4-5):379–401. 10.1080/07494467.2016.1257293

[94] Rocchesso D, Lemaitre G, Susini P, Ternström S, Boussard P. Sketching sound with voice and gesture. ACM Interactions. 2015;22(1):38–41. 10.1145/2685501

