# Supplementary material for: Rising tones and rustling noises: Metaphors in gestural depictions of sounds
Source: PLoS One. 2017 Jul 27;12(7):e0181786. doi: 10.1371/journal.pone.0181786 (PMC5547699; doi:10.1371/journal.pone.0181786)
Supplement: S1 Appendix — (PDF) [file pone.0181786.s001.pdf]

Rising tones and rustling noises: metaphors in gestural depictions of sounds. S1 Appendix

Guillaume Lemaitre[1*], Hugo Scurto[1,2], Jules Françoise[2], Frédéric Bevilacqua[2], Olivier Houix[1], Patrick Susini[1]

1 Equipe Perception et Design Sonores, STMS-IRCAM-CNRS-UPMC, Institut de Recherche et de Coordination Acoustique Musique, Paris, France
2 Equipe Interaction Sons Musique Mouvement, STMS-IRCAM-CNRS-UPMC, Institut de Recherche et de Coordination Acoustique Musique, Paris, France

***GuillaumeJLemaitre@gmail.com**

Analysis grid

Main vocal features	
0	No
1	Noisy component
2	Tonal component
3	Breathing in/out
4	Spectral modulation
5	Roughness
6	Pitch or spectral centroid going up
7	Pitch or spectral centroid going down
Main gestural features	
0	No
1	Sign at the end
2	Arms, hands, or fingers shaking
3	Hands moving inward and outward
4	Hands alternating
5	Fingers closing
6	Fingers opening
7	Both hands getting closer
8	Hands spread or getting apart
9	Movement along a main direction
Direction of the gestures	
0	No
1	Leftward
2	Rightward
3	Upward
4	Downward
5	Outward
6	Inward

Table A. Analysis grid used in the observational study.

Annotations

Sound	0	1	2	3	4	5	6	7
Upward	0	40	90	0	0	10	100	10
Downward	0	30	100	0	0	10	10	100
Refrigerator	0	20	100	0	50	10	20	0
Door	0	90	30	10	40	10	0	70
Stationary	0	100	0	0	30	20	0	0
Scraping	0	100	10	30	80	10	0	0
Filling	0	100	20	0	80	60	60	20
Printer	0	40	100	10	10	20	70	30

Table B. Percentage of vocal features reported by the annotators for the referent sounds in the observational study, averaged across imitators. The numbers at the top correspond to the gestural features reported in Table A.

Sound	0	1	2	3	4	5	6	7	8	9
Upward	0	10	0	0	0	20	40	20	30	100
Downward	0	10	20	0	0	30	20	10	20	100
Refrigerator	0	10	80	30	10	0	0	0	0	40
Door	0	0	20	0	0	0	10	0	10	90
Stationary	0	0	80	0	20	0	10	10	20	30
Scraping	0	0	0	100	10	0	10	0	0	20
Filling	0	20	60	0	0	10	0	30	30	90
Printer	0	0	40	90	30	10	0	0	0	100

Table C. Percentage of gestural features reported by the annotators for the referent sounds in the observational study, averaged across imitators. The numbers at the top correspond to the gestural features reported in Table A.

Sound	0	1	2	3	4	5	6
Upward	0	20	20	100	20	40	10
Downward	10	0	20	40	70	20	10
Refrigerator	60	30	30	0	0	10	10
Door	0	60	0	40	10	20	10
Stationary	60	10	20	0	20	0	0
Scraping	0	50	50	30	30	40	40
Filling	20	10	10	60	30	0	0
Printer	0	40	40	80	80	30	30

Table D. Percentage of directional features reported by the annotators for the referent sounds in the observational study, averaged across imitators. The numbers at the top correspond to the gestural features reported in Table A.

Co-occurrences

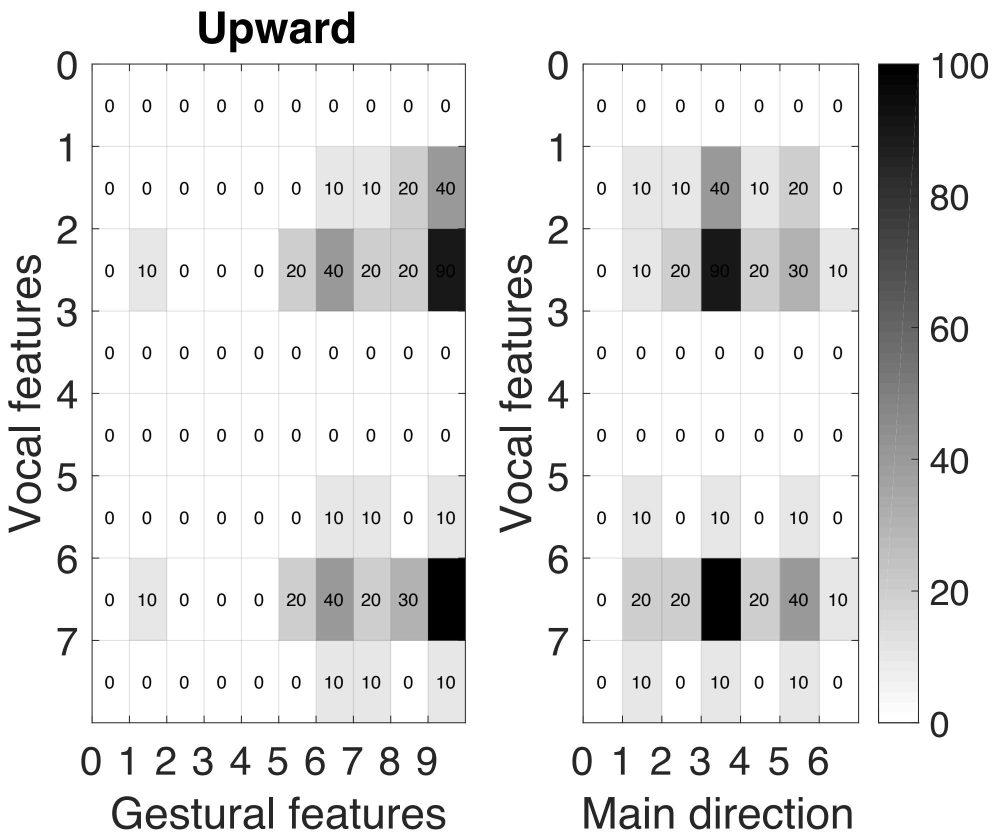

Figure A. Observational study. Co-occurrences of vocal and gestural features (left panel) and vocal and directional features (right panel) for “Upward sweep”. Numbers and gray shadings represent the percentage of co-occurrences. The numbers on each axis correspond to the gestural features reported in Table A.

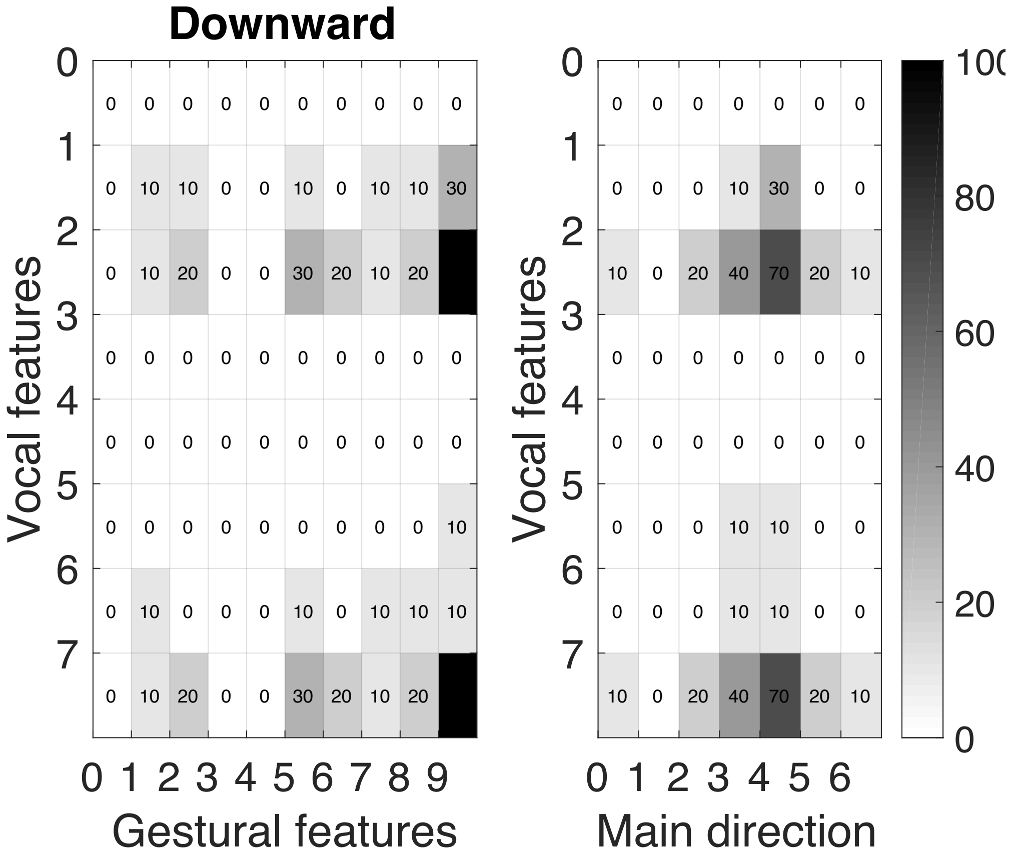

Figure B. Observational study. Co-occurrences of vocal and gestural features (left panel) and vocal and directional features (right panel) for "Downward sweep".

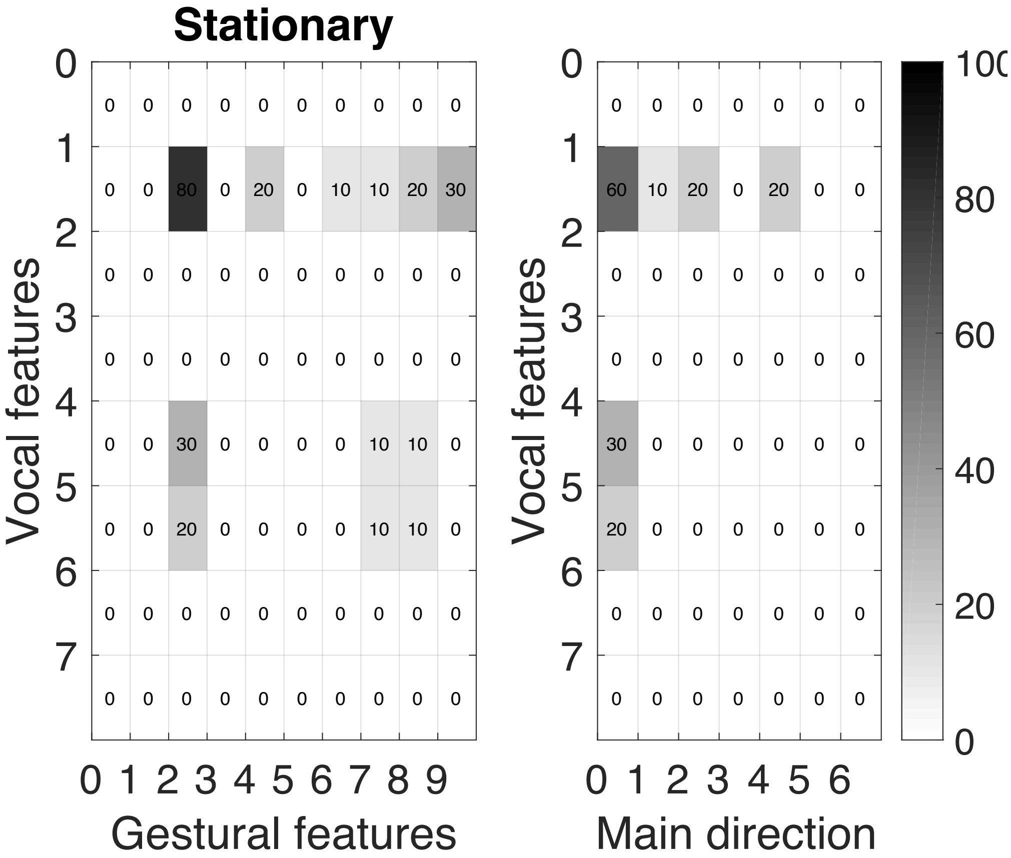

Figure C. Observational study. Co-occurrences of vocal and gestural features (left panel) and vocal and directional features (right panel) for "Stationary noise".

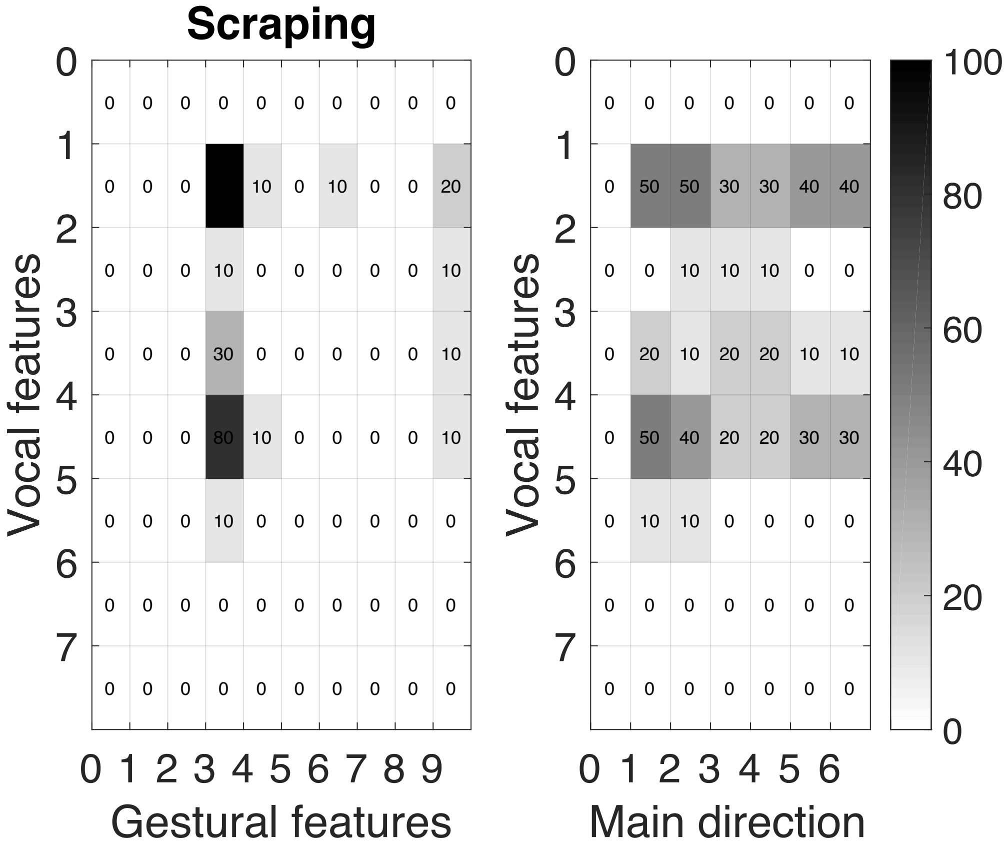

Figure D. Observational study. Co-occurrences of vocal and gestural features (left panel) and vocal and directional features (right panel) for "Scraping".

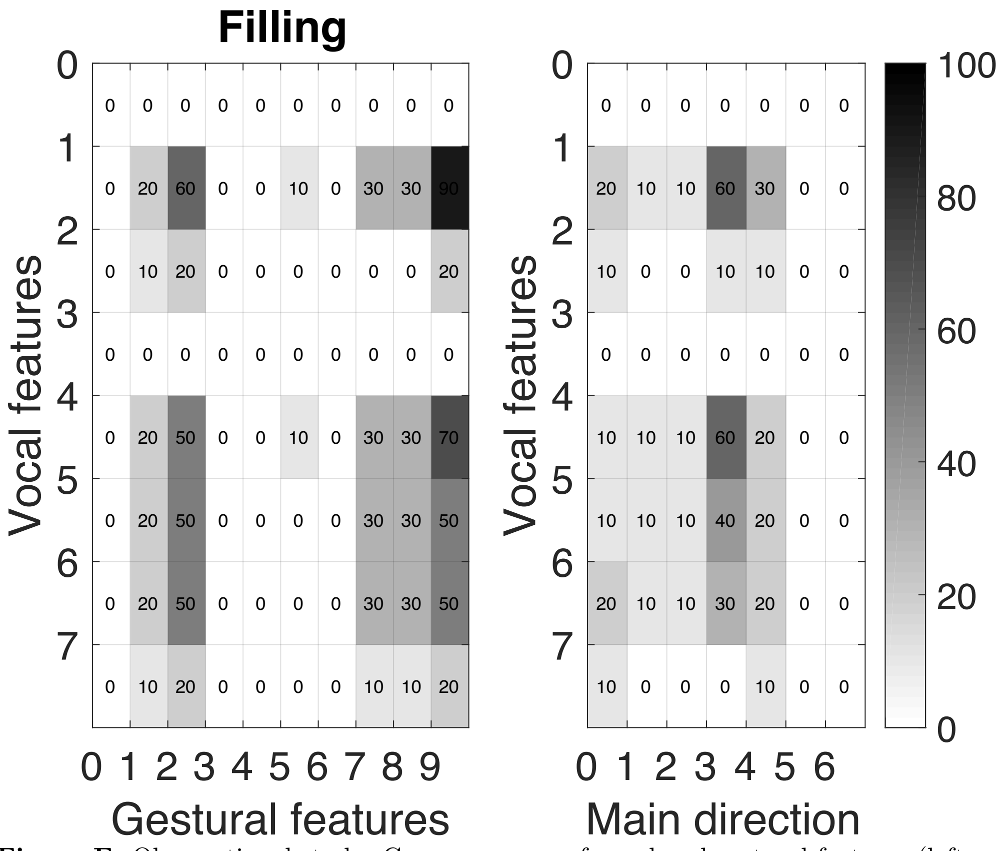

Figure E. Observational study. Co-occurrences of vocal and gestural features (left panel) and vocal and directional features (right panel) for "Filling a receptacle".

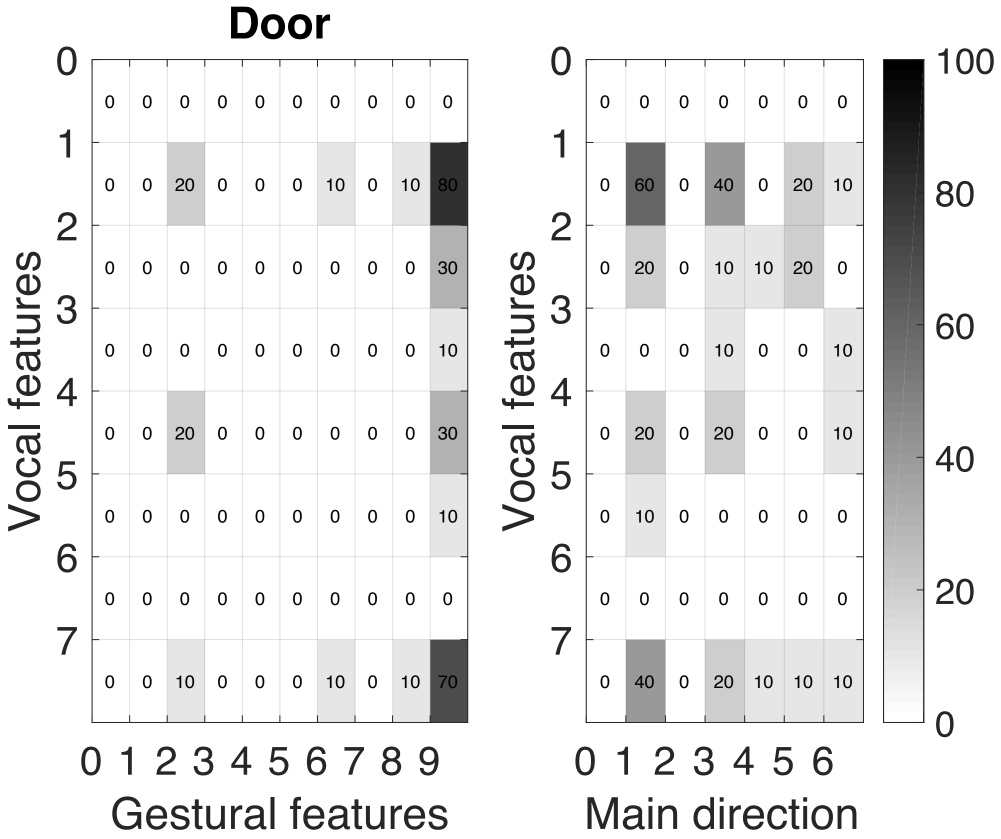

Figure F. Observational study. Co-occurrences of vocal and gestural features (left panel) and vocal and directional features (right panel) for “Door”.

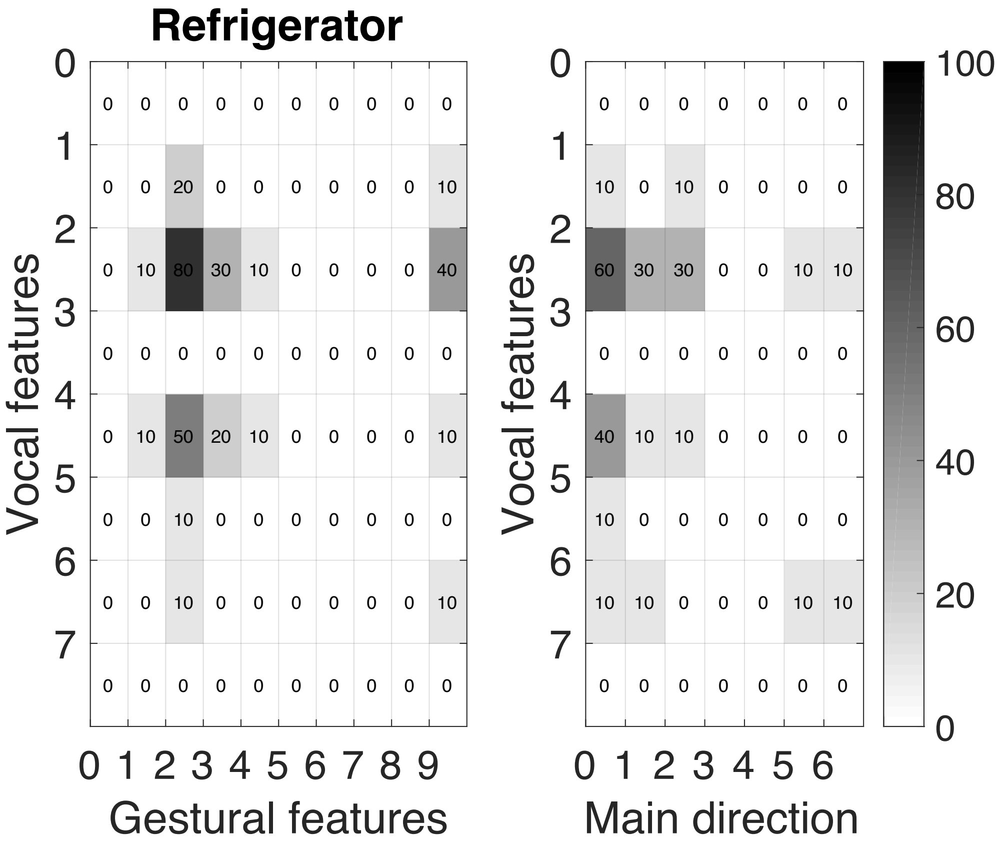

Figure G. Observational study. Co-occurrences of vocal and gestural features (left panel) and vocal and directional features (right panel) for "Refrigerator".

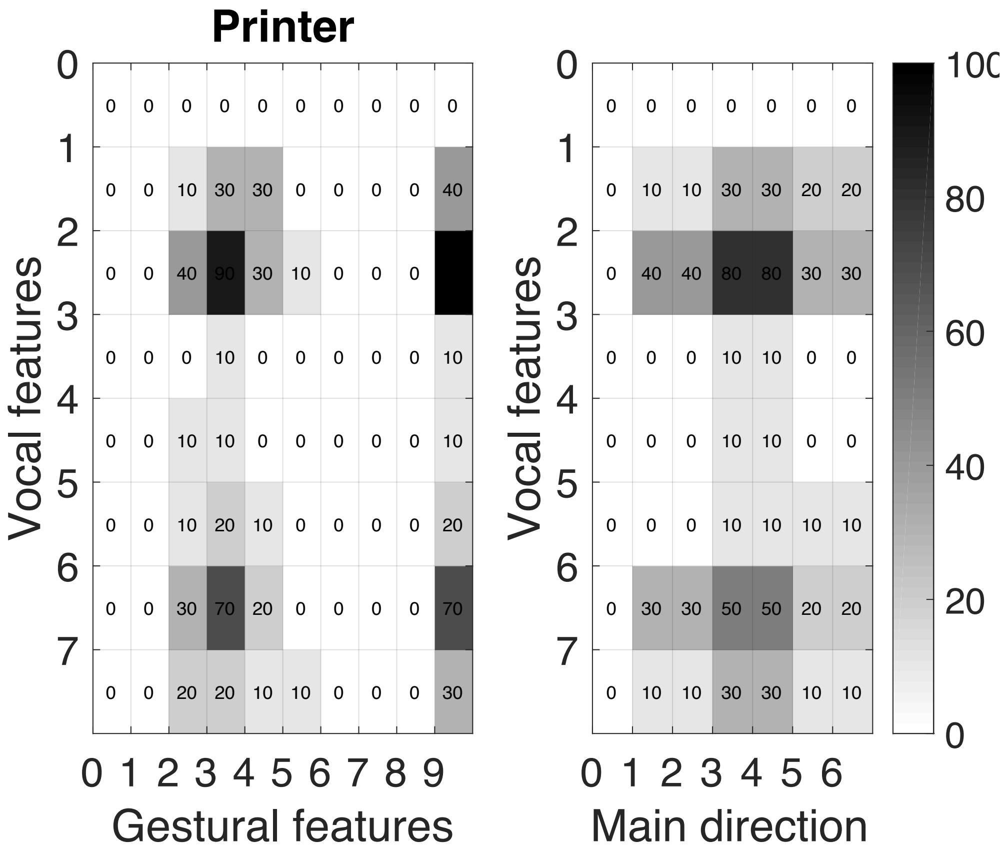

Figure H. Observational study. Co-occurrences of vocal and gestural features (left panel) and vocal and directional features (right panel) for "Printer".
